# Kssd: sequence dimensionality reduction by k-mer substring space sampling enables real-time large-scale datasets analysis

**DOI:** 10.1186/s13059-021-02303-4

**Published:** 2021-03-16

**Authors:** Huiguang Yi, Yanling Lin, Chengqi Lin, Wenfei Jin

**Affiliations:** 1 Shenzhen Key Laboratory of Gene Regulation and Systems Biology, School of Life Sciences, Southern University of Science and Technology, Shenzhen, 518055 Guangdong China; 2 Institute of Life Sciences, Southeast University, Nanjing, 210096 Jiangsu China

**Keywords:** Sketching method, Distance estimation, Sequence comparison, K-mer, Mislabeling detection

## Abstract

Here, we develop k -mer substring space decomposition (Kssd), a sketching technique which is significantly faster and more accurate than current sketching methods. We show that it is the only method that can be used for large-scale dataset comparisons at population resolution on simulated and real data. Using Kssd, we prioritize references for all 1,019,179 bacteria whole genome sequencing (WGS) runs from NCBI Sequence Read Archive and find misidentification or contamination in 6164 of these. Additionally, we analyze WGS and exome runs of samples from the 1000 Genomes Project.

**Supplementary Information:**

The online version contains supplementary material available at 10.1186/s13059-021-02303-4.

## Background

When this manuscript is drafting, the size of NCBI Sequence Read Archive (SRA) database has reached to 30 peta base pairs and is keeping exponential growth [1]. Such volume of data makes conventional sequence analysis tools such as Blast [2] unscalable and puts a heavy burden for data managements and operations. The “big data” challenge was largely alleviated recently by sketching techniques—a class of techniques about how to map *k*-mers (*k* length substrings of a sequence) to integers and select *k*-mers for similarity—or distance-preserving sequence dimensionality reduction [3, 4]. Sketching techniques create a sketch (namely, collection of integers mapped from selected *k*-mers) for each dataset (hereinafter, refers to either a sequence or a sequences set) and use merely sketches that typically are only several Kb in sizes to measure the extents of two kinds of relationships—resemblance and containment, which capture the notions of “roughly the same” and “roughly contained” for two datasets, respectively [5]. Resemblance and containment measurements underlie a broad range of applications, e.g., clustering of datasets, composition analysis of metagenomics, and searching large-scale datasets for a certain sequence [3, 5]. Notably, sketching techniques also play an essential part in long-reads assembly [6] and mapping [7, 8] by overcoming the computational bottleneck of all vs. all long-reads overlapping and long-reads to reference mapping, respectively.

Current sketching techniques select *k*-mers by minimum hashes, hence also termed as MinHash methods. For example, Mash [3] and Bindash [4] select fixed number (typically around 1000) of *k*-mers with minimum hashes of all the *k*-mers from each dataset, for the reduced representation of this dataset. MinHash methods have inherited limitations from hash: Hash is a many-to-one function, meaning *k*-mers hashing is irreversible and distinct *k*-mers could be mapped to the same integer, namely, hash collisions. Hash collisions would introduce extra variation to the resemblance or containment measurement and thereby may hamper the accuracy for datasets searching, distance estimation, and clustering. Moreover, MinHash methods such as Mash and Bindash always create sketch of fixed size, which would lead to a biased distance estimation when comparing two datasets of very different sizes [3, 5]—as is the scenario of many potential applications, e.g., prioritizing the references for a metagenomics dataset or composition analysis of a mixture of sequences dataset. Mash screen [9] was recently developed to address the latter problem; it first sketches all the references, then query *k*-mers from each mixture of sequences against the reference sketches to calculate the proportion of *k*-mers of each reference contained in each mixture of sequences. But the mixture of sequences which consume majority of space are not sketched at all; hence, the merits of sketching techniques—extremely high space and time efficiency—are largely compromised. It makes mash screen more suitable for answering the question “what reference genomes contained in my (one) run?” [9], but not the question “what reference genomes contained in which runs?”

**Fig. 1:**
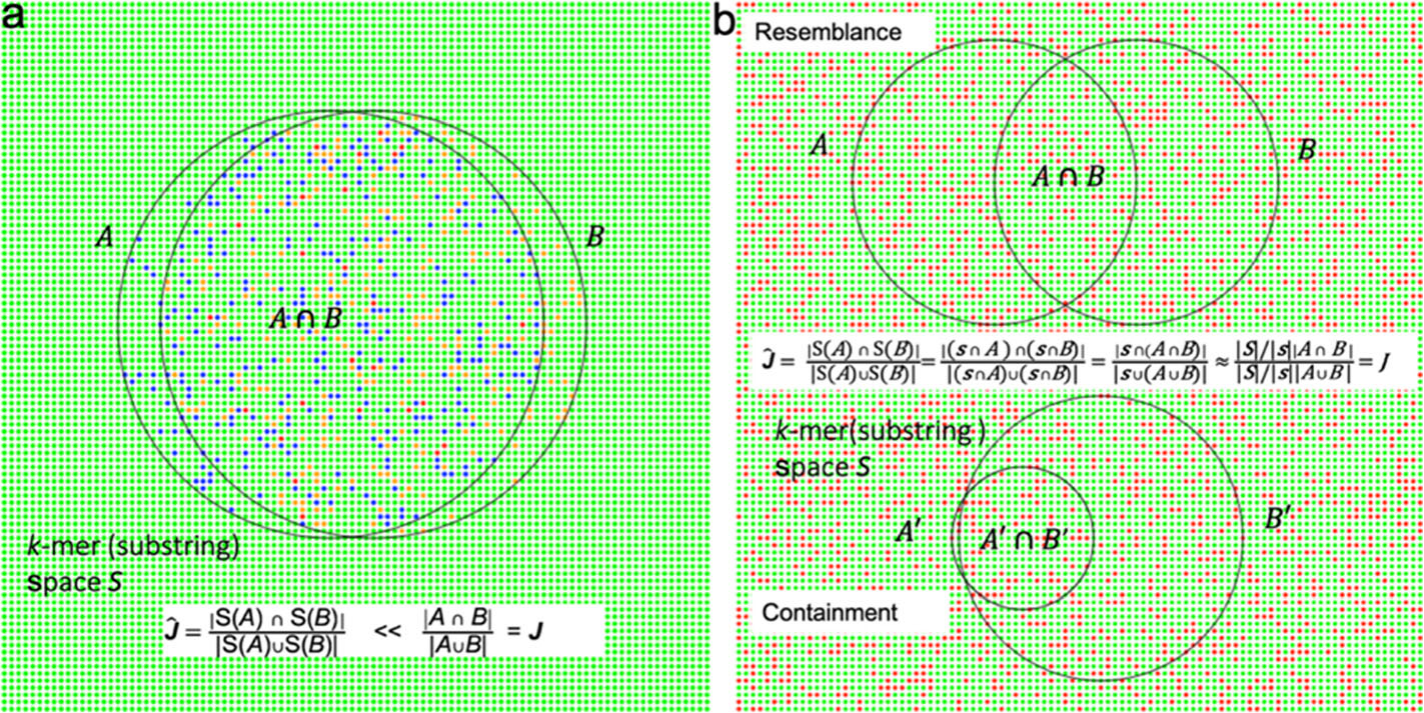
The main idea of Kssd. The Kssd idea originates from the naive sketching method of sampling *k*-mers directly from the sequence as its sketch as illustrated in **a**: the *k*-mers randomly drawn from sequence *A* and *B* are represented by blue dots *S*(*A*) and orange dots *S*(*B*), respectively, and the shared *k*-mers *S*(*A*) ***∩*** *S*(*B*) are represented by red dots. However, such a sketching method is ill-suited for similarity (or distance) estimation since the two sketches *S*(*A*) and *S*(*B*) are probably drawn from unrelated regions of *A* and *B* hence shared very few *k*-mers (with an estimated Jaccard coefficient $\hat{\boldsymbol{J}}$ approximate to 0) even when *A* and *B* are nearly identical. Notwithstanding its naivety, this thought inspired us the idea of *k*-mer space sampling as illustrated in **b**: firstly, a subset of *k*-mers *s* termed *k*-mer subspace (shown as red dots here) are drawn randomly from *k*-mer space *S* (namely the collection of all possible string of length *k* defined in a given alphabet set, shown as green dots **∪** red dots here); then, the sketch of any given sequence is built by overlapping *s* with the *k*-mers set of this sequence. Since *s* is an unbiased sampling of the *k*-mer space *S*, it is independent of any instance *k*-mer sets. After sketching, two sequences *A* and *B*, their intersection *A* **∩***B* and union *A* ***∪*** *B* should go through dimensionality reductions of the same expectation fold of $\frac{\mid \boldsymbol{S}\mid }{\mid \boldsymbol{s}\mid }$. Therefore, it enables measuring both the resemblance and the containment of the two sequences directly using their sketches, even if they are of very difference sizes

To better address the above question as well as the hash collisions problem, we propose here a new sketching method named *k*-mer substring space decomposition (Kssd) [10, 11]. The main idea of Kssd is based on *k*-mer space sampling: a set of *k*-mers are first drawn by random from the whole *k*-mer space and then overlapped with each given dataset to create the sketch (Fig. 1). The sketches created this way could preserve the pairwise similarities (or distances) of given datasets even when the datasets are of very different sizes (see the “Methods” section for proofs). *k*-mer space sampling is generalized to *k*-mer substring space sampling/shuffling permitting sampling from a much smaller space so that computational cost could be dramatically reduced. Kssd avoids hash collisions using a pre-determined *k*-mer recoding scheme (see the “Methods” section). Such a collision-free feature ensures the accuracy of set operations on sketches. Kssd supports three types of sketch set operations: union, intersection, and subtraction, which mirror respective set operations on original *k*-mers sets and underlie a broad range of potential applications (see the “Discussion” section). Here, we compare Kssd with other sketching methods on computational efficiencies and accuracies using both simulated and real data. We further illustrate the usages of Kssd in (1) creating sketches for all metagenomic datasets, (2) optimal references and species inconsistency detection for all bacteria WGS datasets and (3) human population datasets clustering, mislabeling detection, and correction using reference subtraction (a featured application of Kssd).

## Results

### Kssd algorithm overview

**Fig. 2:**
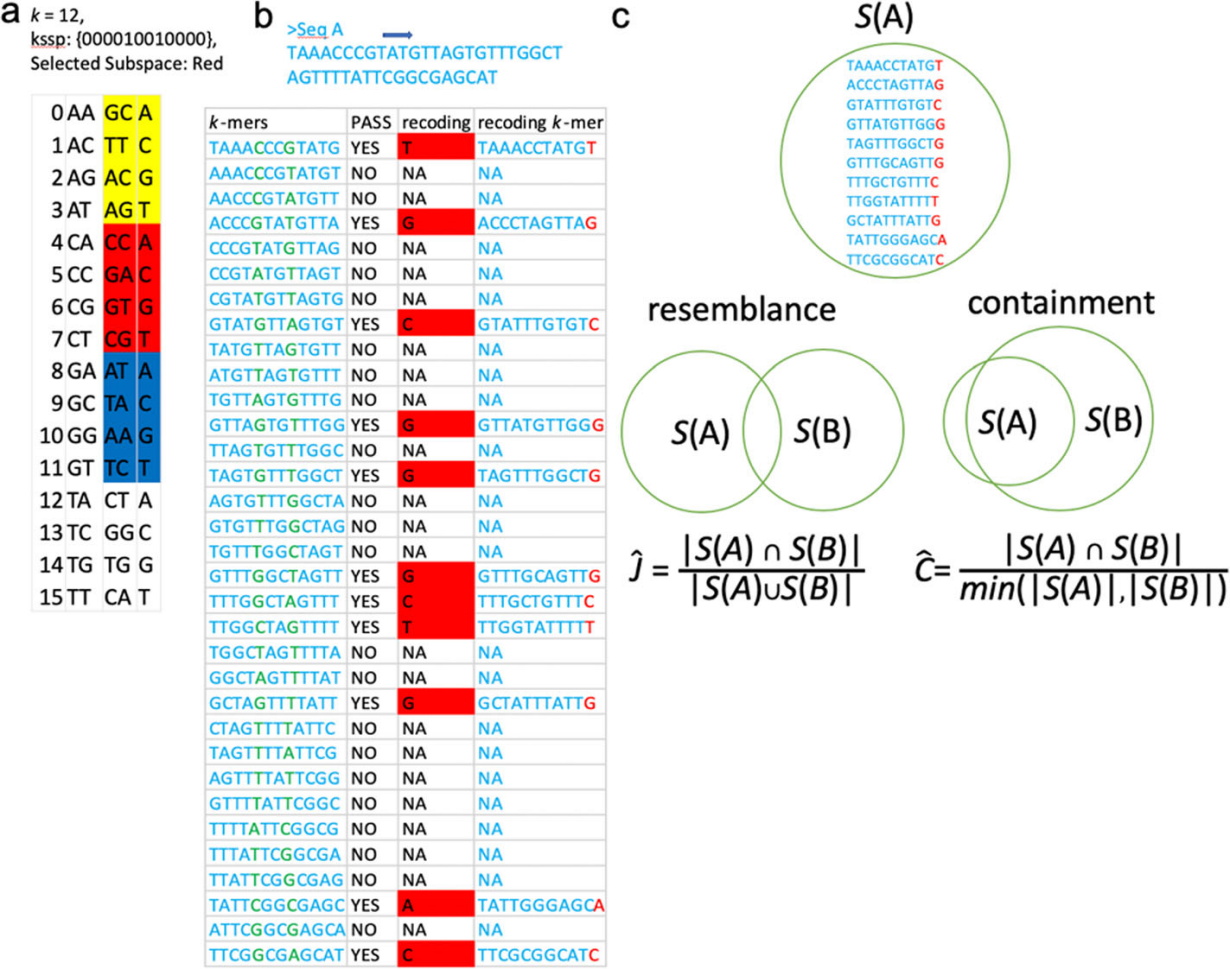
Kssd algorithm overview. **a** *k*-mer substring space shuffling. In this example, a *k*-mer substring selection pattern (kssp) *p* = ‘000010010000’ is pre-determined for 12-mer analysis, so the length of *p*-selected-substring is 2 and the *k*-mer substring space has dimensionality *D* = 4^2^ = 16. This 16-dimensions space is shuffled and partitioned into *N* subspaces of equal size (3rd column, *N* = 4 here), and the dimensions in each subspace are recoded by the lexically ordered strings of length ${\boldsymbol{\log}}_{\mathbf{4}}\frac{\boldsymbol{D}}{\boldsymbol{N}}$ (4th column, length = 1 here). One subspace *s* (3rd and 4th column, marked as red) is chosen for sequence sketching. **b** Sequence sketching. First, the *k*-mers with *p*-selected-substring (green substring in 1st column) belonging to the red subspace *s* are selected, where the *p*-selected-substrings are recoded by the lexically ordered dimension (3rd column), and each selected *k*-mers is recoded in a way that the recoded *p*-selected-substring suffixes the rest substring (4th column). **c** Kssd distance. Once all sequences were sketched, the Jaccard and containment coefficients are estimated by $\hat{J}$ = $\frac{\mid S(A)\mathbf{\cap}S(B)\mid }{\mid S(A)\cup S(B)\mid }$and $\hat{C}$ = $\frac{\mid S(A)\mathbf{\cap}S(B)\mid }{\mathit{\min}\left(|S(A)|,|S(B)|\right)}$, respectively

We first introduce two conceptions: *k*-mer substring selection pattern (kssp) and *k*-mer substring space sampling/shuffling. A kssp (denoted by *p*) is a “01” string of length *k*, where 1s positions of a *k*-mer define the *k*-mer substring (denoted by *p*-selected-substring). The whole *k*-mer substring space is randomly shuffled, partitioned into *N* subspaces of equal size and one of the *N* subspaces is chosen for sequence sketching, so that the dimensionality reduction rate is $\frac{1}{N}$ (Fig. 2a). kssp setting and *k*-mer substring space sampling/shuffling need be performed only if *k*-mer substring space has never been defined (in a file), e.g., run Kssd for the first time. Otherwise, kssd has only two steps: (1) sketching and (2) distance computing as other sketching methods. Sketching: for a given sequence, *k*-mers with its *p*-selected-substring presented in the chosen *k*-mer substring subspace are selected and recoded into sketch (Fig. 2b). Distance computing: once all sequences are sketched, the Jaccard and containment coefficients are estimated by $\hat{J}$ = $\frac{\mid S(A)\cap S(B)\mid }{\mid S(A)\cup S(B)\mid }$and $\hat{C}$ = $\frac{\mid S(A)\cap S(B)\mid }{\mathit{\min}\left(|S(A)|,|S(B)|\right)}$, respectively (Fig. 2c). More detailed characterization of the algorithm and its statistical properties is available in the “Methods” section.

### Accuracy of resemblance estimation

**Fig. 3:**
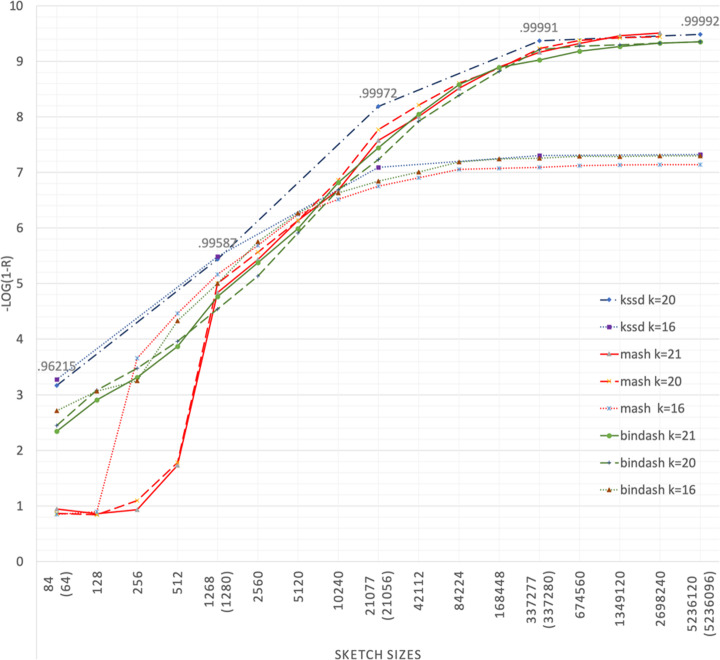
Accuracies of Kssd, Mash, and Bindash on closely related group. The Pearson correlation coefficients *r* between the ground truth and the estimated mutation rates is scaled to **− log** (**1 −** ***r***) for plotting clarity (*y*-axis). The decimal above the highest data point at a sketch size is the maximal *r* value of all the three methods of all *k* settings with that sketch size. The default *k*-mer lengths *k* for Kssd, Bindash, and Mash are 16, 21, and 21, respectively; to match *k*, we also run Mash and Bindash with *k* = 16 in addition to the default *k* settings, but Kssd takes only even *k*, so we also run with *k* = 20 to vary *k*. Due to different sketching mechanism, Mash and Bindash take as parameter the sketch size of continuous integers and multiples of 64, respectively; but sketch size is not a parameter of Kssd and can only counted from the sketch file. To match sketch sizes as closely as possible, we first sketched the reference using Kssd with dimensionality reduction levels *z* = {4, 3, 2, 1, 0} and obtained the sketch sizes *s*_*k*_ = {84, 1268, 21,077, 337,277, 5,236,120}, respectively; we got the nearest multiples of 64 of *s*_*k*_ (the parenthesized values) and interpolated with their 2-, 4-, and 8-fold sketch sizes to obtain the sketch sizes parameter *s*_*b*_ for Bindash; and we merged *s*_*k*_ and the interpolated points of *s*_*b*_ to obtain the sketch size parameter *s*_*m*_ for Mash. Mash with *k* = 20, 21 at sketch size 5,236,120 are not shown due to the running error

Resemblance is the relationship that captures the distance/similarity of two datasets of similar sizes. To compare the accuracy of Kssd with other sketching methods on resemblance estimation, we evolved in-silico a reference genome to 600 mutated genomes with pre-defined mutation rates ranging from 0.001 to 0.60 with stepwise increase of 0.001 which were served as ground truths hereinafter. Mutated genomes were divided into closely related group and distantly related group, with mutation rate being 0.001~0.3 (Fig. 3) and 0.301~0.60 (Additional file 1: Fig. S1), respectively. The mutation distances between reference and its mutants were estimated by Kssd, Mash, and Bindash, with variated *k*-mer lengths and sketch sizes. Pearson correlation coefficient of the ground truths and the estimations was adopted as the accuracy measurement.

The results show clear stratifications with respect to *k*-settings, where *k* = 20 and 21 yield generally similar curves whereas *k* = 16 yield alternative curves. When sketch size is relative smaller, using shorter *k* (16 here) is more accurate than longer *k* (20, 21 here); but when sketch size is larger (larger than 10,240 and 42,112 in closely related group and distantly related group, respectively), using longer *k* is more accurate (Fig. 3 and S1). However, for the sake of efficiency, sketching technique rarely use sketch size larger than 10,000, so shorter *k* is preferred. Providing the same *k*-settings (*k* = 16 or 20), Kssd outperforms both Mash and Bindash on both groups, especially when sketch size is small (84, 1268 and 21,077) where Kssd’s accuracy is on par with Mash/Bindash’s with double sketch size. For applications requiring an accuracy threshold of distance estimations, it means kssd can use 2 times higher dimensionality reduction for sketching, hence achieving 2 times less space and time costing than Mash/Bindash.

### Comparisons of Kssd and Mash screen on containment estimation and metagenomic datasets sketching

Containment is the relationship that captures the distance/similarity of two datasets of very different sizes. As mentioned above, the shortcomings of Mash and Bindash on containment estimation are obvious and the best reported method for containment estimation is Mash screen [9]. To compare Kssd with Mash screen, we benchmarked Kssd by the same approach used by Ondov et al., where a constitutes-known synthetic microbiome community (accession: SRR606249; referred as shakya dataset [12]) was adopted as testing dataset. Shakya dataset contains also exogenous genomes due to sample contaminations which could be revealed by comparing to the simulated reads from the 64 known constitutes (served as the contamination-free control) [9].

**Fig. 4:**
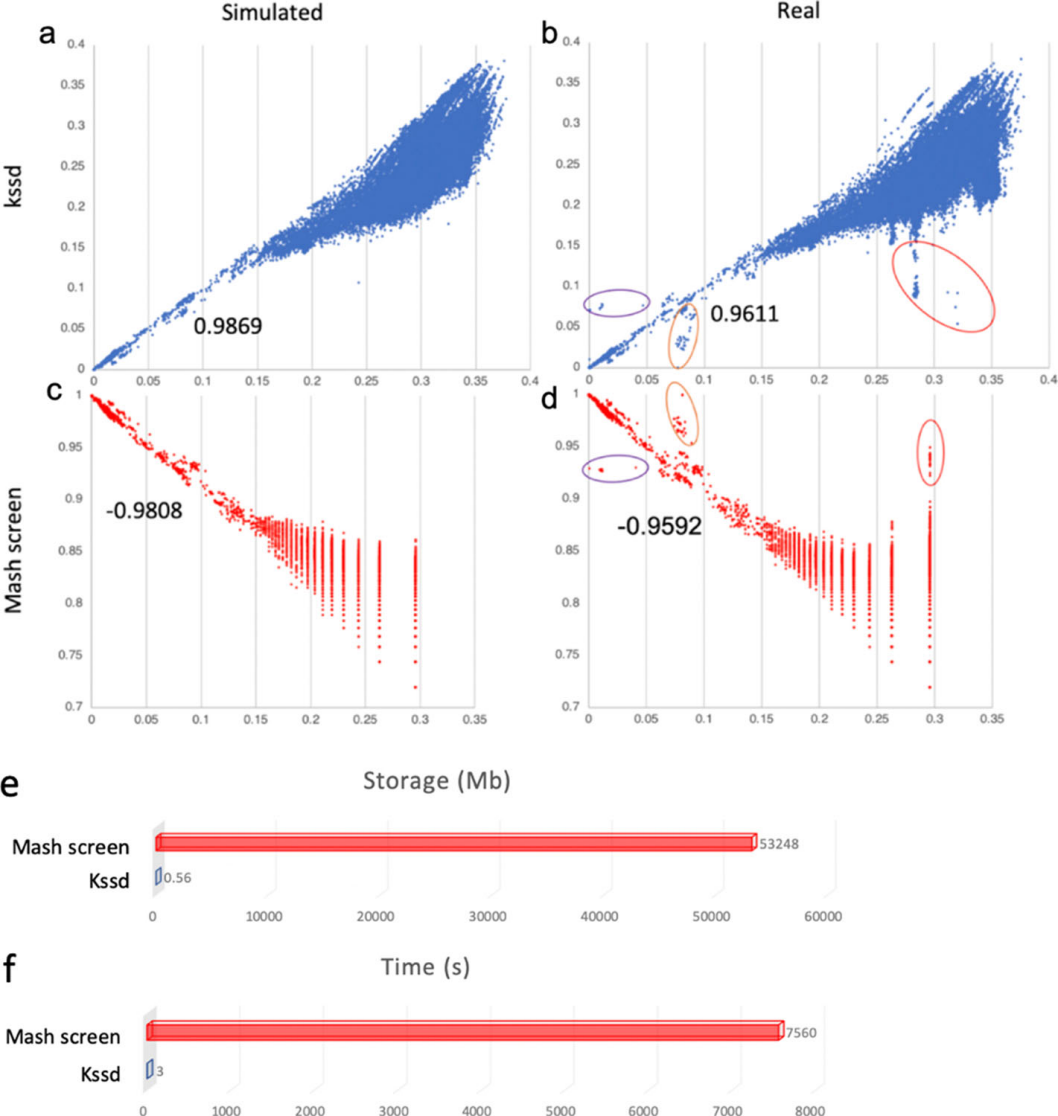
Comparisons of Kssd and Mash screen on containment estimation using shakya datasets. **a**–**d** Each dot represents a reference. *X*-axis indicates the minimum resemblance distance (Eq. 4) from a reference to the 64 known constitutes computed by Kssd (**a**, **b**) or Mash (**c**, **d**). *Y*-axis indicates reference-to-mixture containment measurements computed by Kssd (using Aaf distance [13], Eq. 5) and Mash screen (containment-score [9]). Containment score (*y*) of Mash screen measures the similarity between reference and mixture; hence, it negatively correlated with *x*. The decimals below the data points are the correlation coefficients (*r*) of the plot controlling *x* < 0.15. The 1st and 2nd columns are the plots of the simulated and the real shakya datasets, respectively. On the real shakya datasets, the data points biased from expectation due to low abundance constitutes are circled in purple, and those biased due to two different contaminations are circled in orange and red. **e** Suppose remote database adopt Kssd sketching and provide Kssd sketch for shakya dataset, the user can greatly reduce their storage costing when performing this analysis. **f** For large-scale containment analysis, where the sample size of sequences mixture is greatly larger than the number of references, the asymptotic time consumption of Kssd is much smaller than that of Mash screen

We took the latest NCBI prokaryotic assembly (including 138,743 genomes) as references. For each reference, the containment measurements of this reference to shakya datasets were plotted against the minimum resemblance distance (Eq. 4) of this reference to the 64 known constitutes (hereinafter referred as minimum reference-to-constitutes distance). Kssd containment analysis conveys almost the same information with that of Mash screen, where data points were biased due to the same low-abundance constitutes and the same contaminations (Fig. 4a–d). Data points from Mash screen became increasingly discrete along *x* and *y* that probably due to using fixed sketch size, whereas those from Kssd kept continuous which is more realistic. Both Kssd and Mash screen containment measurements were highly correlated with minimum reference-to-constitutes distances less than 0.15 in both the simulated (|*r*| = 0.9869 versus 0.9808) and the real (|*r*| = 0.9611 versus 0.9592) shakya datasets (Fig. 4a–d).

Notably, the Kssd sketch for shakya datasets was only 580 Kb. Suppose a remote database (e.g., NCBI) manages to adopt kssd sketching and provide kssd sketches for the raw datasets, users could spend 110,535 times less storage cost and data transfer for containment analyses using kssd than using mash screen that takes the raw shakya dataset (52 Gb, Fig. 4e). Moreover, Kssd took only 23.5 CPU minutes for the raw dataset sketching and 3 CPU seconds for the containment estimation, whereas Mash screen took in total 126 CPU minutes for screening and the containment estimation. There is no need for Kssd to sketch the shakya dataset again for the containment analysis for new references whereas Mash screen would take another 126 CPU minutes to screen the shakya dataset again for another 138,743 new references, which is likely happen in the near future since NCBI Refseq database is growing rapidly [14]. When performing containment analysis of all SRA datasets (> 1 million) against all new references (here, 138,743), the sketching time for references is neglectable; therefore, the asymptotic time consumption of Kssd is only $\frac{1}{\ 2523}$ of that of Mash screen (3 CPU seconds vs. 126 CPU minutes, Fig. 4f). Such a high storage and time efficiency makes kssd well-suite for answering the above-mentioned question “what reference genomes contained in which runs?”

Encouraged by the computational efficiency of Kssd, we applied Kssd to sketch all metagenomic datasets in SRA. Although all metagenomics containment analysis has been conducted using Mash screen before [9], Mash screen cannot generate sketches of the metagenomic datasets for future analysis. Here, we created their sketches using Kssd. Total 190,518 metagenomics runs were retrieved in SRA database. However, due to frequent failures on prefetch and fastq-dump, 65,265 runs were successfully piped to Kssd sketching (at 4096-fold dimensionality reduction, with minimum *k*-mer occurrence of 2), resulting in a combined sketch of only 10 Gb in size. The user can quickly conduct the containment analysis on their own using the metagenomic combined sketch and the prokaryotic Refseq combined sketch (Additional file 4).

### Computational efficiency

**Fig. 5:**
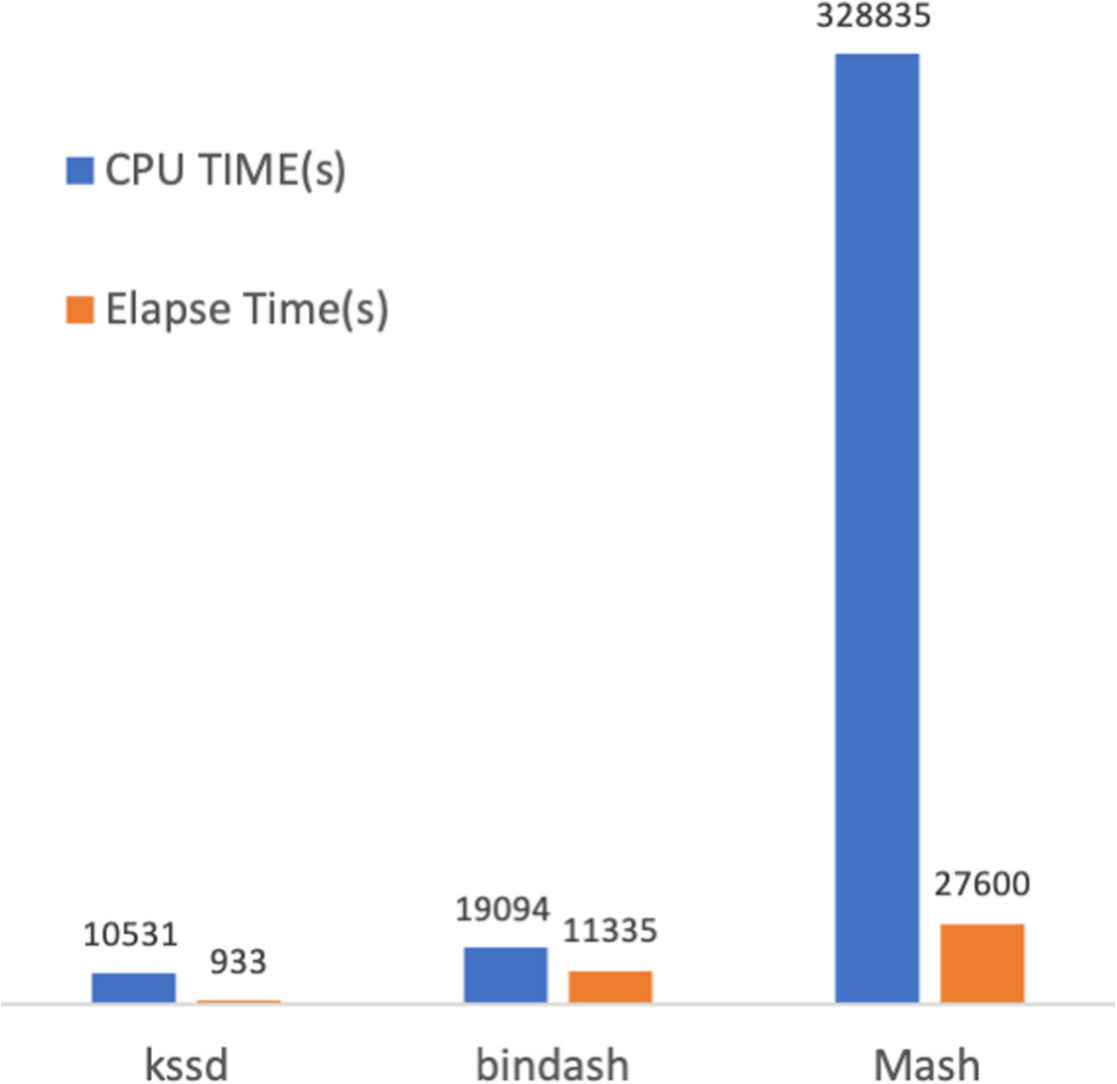
Computational efficiencies. Assessments of CPU time and elapse time of the three methods were performed on a 32-Gb, 12-core machine using a test dataset consisting of 100 K bacteria genomes

We used 100,000 bacteria genomes to assess the time efficiency of distance computing for Kssd, Mash, and Bindash. Both Kssd and Bindash cannot fine-tune the sketch sizes, Kssd take sketch sizes of $\frac{1}{16^z}$ of the size of origin *k*-mers set, and Bindash takes sketch sizes of 64 *m*, where *z*, *m* ∈{0, 1, 2, 3, …}. Here, we choose sketch sizes as close as possible (1780 and 2048 for Kssd/Mash and Bindash, respectively). In our 12-core testing machine, it took 10,531, 19,094, and 328,835 CPU seconds for Kssd, Bindash, and Mash, respectively, and took 933, 11,335, and 27,600 elapsed seconds for Kssd, Bindash, and Mash, respectively (Fig. 5) to complete all pairwise distances computation. Namely, Kssd was 12 times and 30 times faster than Bindash and Mash respectively when using same number of threads (12 threads here). Kssd is significantly speeded up relative to Bindash when multithreaded (from 2 times to 12 times); this may due to a more optimized parallel design or a better I/O characteristics of Kssd than Bindash. Though Bindash used a bit larger sketch size, Kssd can achieve similar accuracies by using only a half of the sketch sizes of Mash/Bindash (Fig. 3), implying Kssd may outperform Mash/Bindash even more with same accuracy.

### Reference prioritization and species inconsistency detection for all bacteria WGS datasets

We then applied Kssd for containment analysis for all bacteria whole genome sequencing (WGS) runs on NCBI SRA database to find the optimal references for each run and to identify those runs in which the species best contained is not the species it registered (hereinafter, runs of species inconsistency). Species inconsistency indicates possible sample contamination or mislabeling which should be examined for quality control purpose. And it also implies possible species misidentification (e.g., by a low resolution method like 16S rRNA), detection of which is particularly important for clinical pathogen datasets since it may help preventing possible misdiagnosis and mistreatment for clinical infectious disease. Though other methods like Mash (screen) may also be used for this task, Kssd is a more suitable tool due to its higher computational efficiency as shown above.

**Fig. 6:**
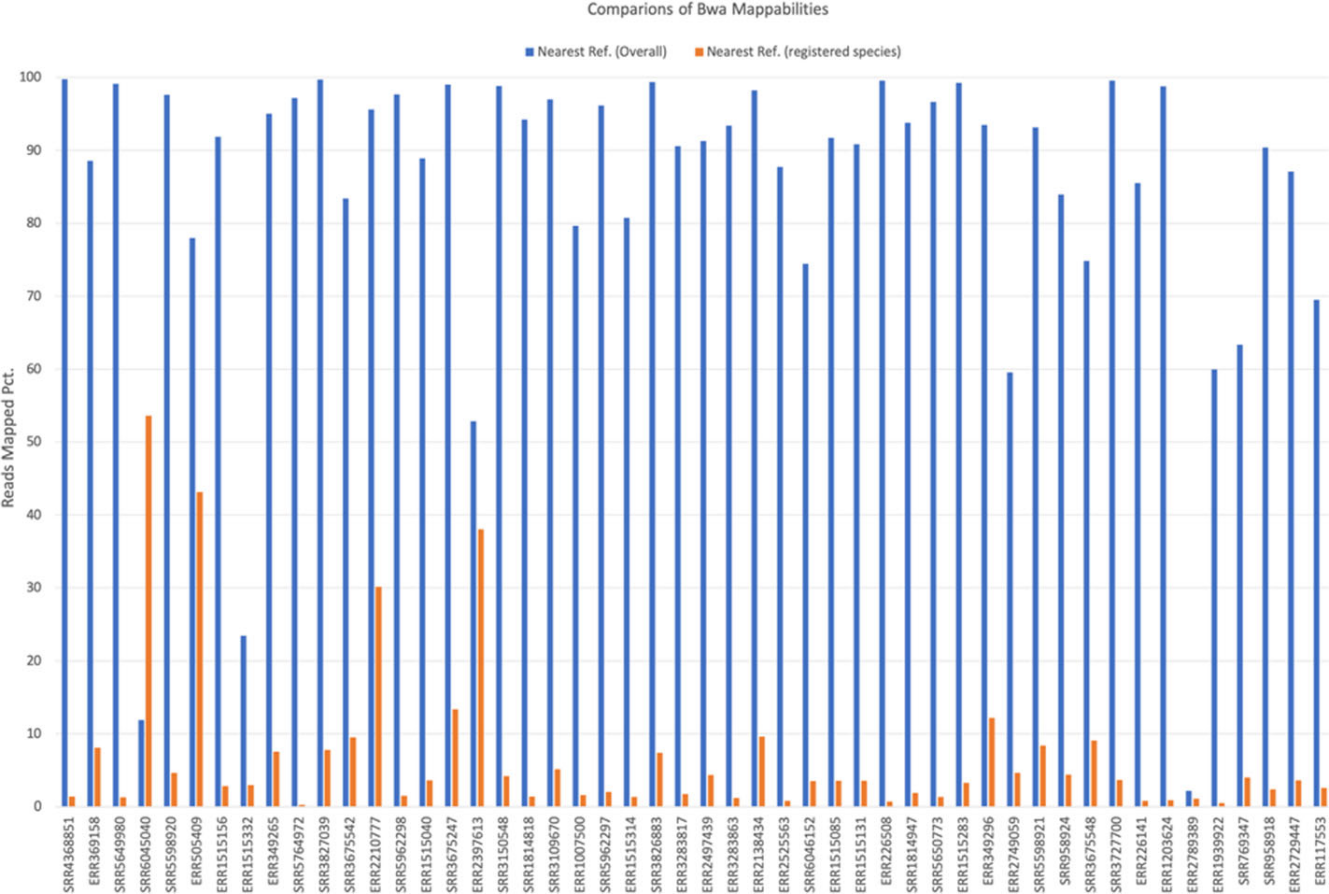
Bwa mappabilities of the 50 sampled runs on the overall nearest reference versus the nearest reference of registered species. Horizontal axis indicates the run accession; vertical axis indicates the percentage of reads bwa mapped to the overall nearest reference (blue) and the nearest reference of registered species (orange)

We first retrieved all bacteria runs on NCBI SRA browser by specifying the organism field to be “bacteria”; from the returned run summary, we excluded those metagenomics and non-WGS runs, resulting in 1,023,960 bacteria WGS runs with total size of 1.4 Pb. 1,019,179 runs were successfully fastq-dumped for Kssd sketching at 4096-fold dimensionality reduction with minimum *k*-mer occurrence of 2, resulting in a combined sketch of only 6.7 Gb in size, which is more than 200,000-fold size reduction relative to the raw datasets. The all-runs sketch was queried against the all-prokaryotic-Refseq sketch, which yielded 141,403,951,997 containment coefficients and Aaf distances (Eq. 5 in the “Methods” section) in only 3 h 22 min on a 12-CPU machine. For each run, 10 references with highest containment coefficients (or minimum Aaf distances) were selected as the optimal references (Additional file 4). Runs of species inconsistency were identified based three criteria: (1) the run had an Aaf distance greater than 0.05—the evidenced upper boundary of intra-species mutational distance [3, 15]—to the nearest reference of the registered species; (2) the run had an Aaf distance less than 0.01 to the overall nearest (namely, best contained) reference; and (3) the overall nearest reference was of a different species from the registered one. In total, 6164 runs of species inconsistency were identified (Additional file 2: Table S1), 50 of which were sampled for validation as follows: for each run, the reads were bwa [16] aligned respectively to the overall nearest reference and the nearest reference of the registered species, and the percentages of reads mapped were calculated and compared. It showed 49 of the 50 runs had higher percentages of reads mapped on the overall nearest reference than on its counterpart (Fig. 6), which suggested that even if a dataset had a wrong registered species, Kssd can still find the optimal reference for the dataset in 98% cases. The only outlier SRR6045040 has lower reads mappability on the overall nearest reference than on the registered species reference. A possible explanation for this could be that the overall nearest reference was poorly assembled which hampered the reads mapping. Although we had excluded metagenomics runs, there were still substantial runs of species inconsistency contained more than one species or unknown species (e.g., ERR2789389), which may indicate severe sample contamination. Otherwise, runs of species inconsistency contained only one dominative species indicated possible species misidentification or mislabeling.

### Population dataset clustering and mislabeling detection and correction using Kssd reference subtraction

**Fig. 7:**
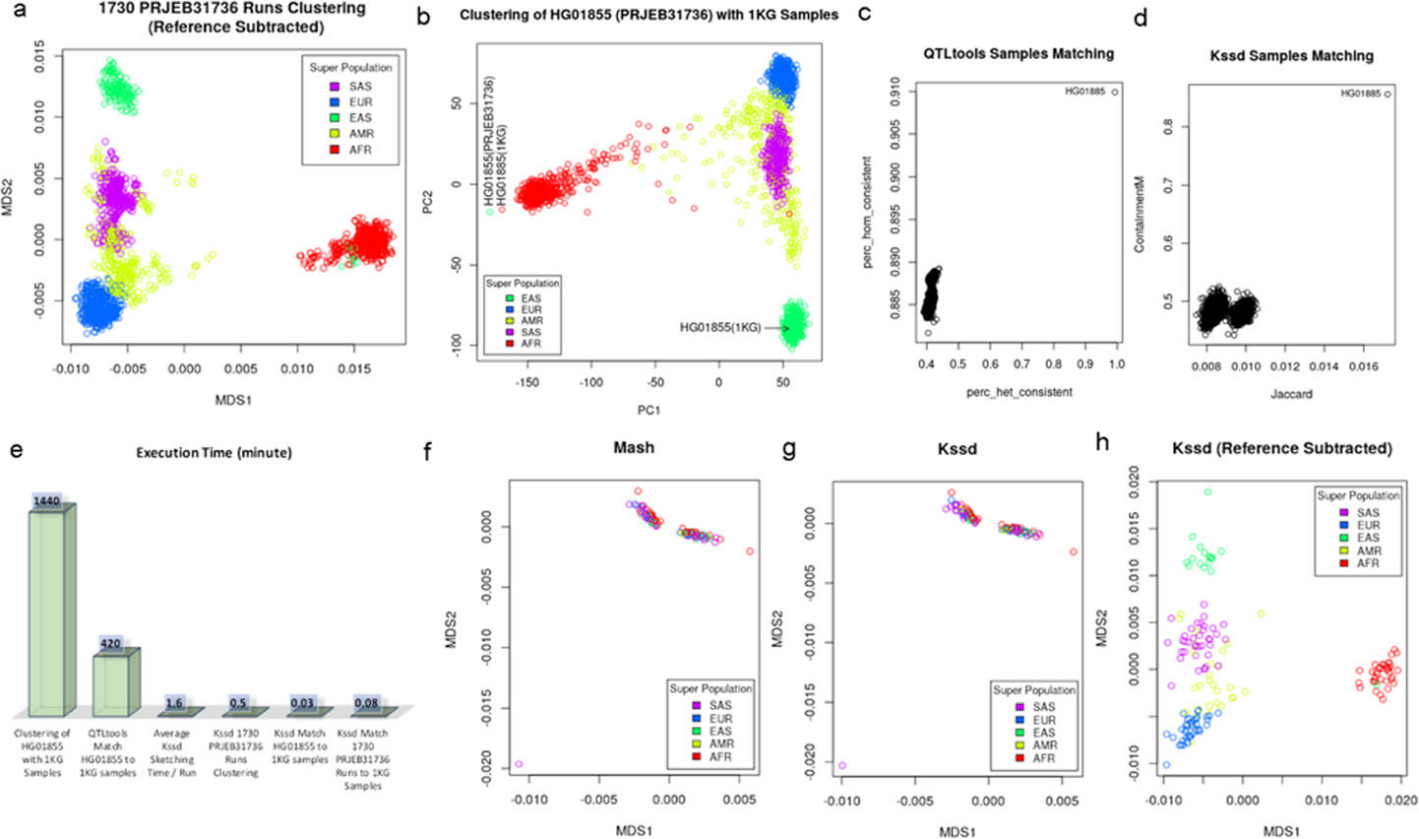
Clustering and matching PRJEB31736 runs by referring 1KG samples. **a** MDS plot of 1730 PRJEB31736 runs using Kssd reference subtracted sketches. **b** Genotype PCA using combined VCF file of HG01855 (PRJEB31736) and 2504 1KG samples. **c** Matching HG01855 (PRJEB31736) to 2504 1KG samples using QTLtools mbv. Each black circle represents a 1KG sample, with *x* and *y* indicating percentages of consistent genotypes on heterozygous and homozygous sites, respectively. **d** Matching HG01855 (PRJEB31736) to 2504 1KG samples using Kssd. Each black circle represents a 1KG sample, with *x* and *y* indicating Jaccard and containment coefficients to HG01855, respectively. **e** Execution time of the above analyses. MDS plot of 160 testing runs using **f** Mash sketches, **g** Kssd sketches without reference subtraction, and **h** Kssd sketches with reference subtraction

As far as we know, Kssd is the only sketching method supports secure subtraction operation between two sketches (see the “Methods” section), which could be particularly useful for population datasets analyses, since those *k*-mers covering the variants—informative sites of population genomics—could be enriched by subtracting the *k*-mers set (or sketch) of reference from that of a given dataset. We used PRJEB31736—an ongoing 30X whole genome resequencing project for the 2504 samples of 1000 Genomes Project phase 3 (hereinafter, 1KG)—to test the performance of Kssd with reference subtraction (hereinafter, Kssd-w/-rs). In total 1730 runs from 196 samples were successfully fastq-dumped for Kssd sketching at 4096-fold dimensionality reduction, with minimum *k*-mer occurrence of 2. All the sketches were subtracted by the sketch of hg38 human reference, resulting in 1730 remainder sketches with total size of just 46 Mb. The pairwise distances of the remainder sketches then were estimated using Kssd and plotted using multidimensional scaling (MDS). It showed the majority of the runs were grouped unambiguously by populations, except that all the runs (ERR3576658, ERR3576659, ERR3576661-ERR3576667) from an EAS sample HG01855 were grouped together with AFR runs (Fig. 7a). The population inconsistency of sample HG01855 implied an AFR sample (real label unknown yet) was mislabeled as HG01855 (an EAS sample) in PRJEB31736; otherwise, Kssd-w/-rs might be not reliable for population-level clustering. To confirm this mislabeling event and justify the Kssd-w/-rs clustering result, we preformed conventional genotypes principal component analysis (PCA) clustering analysis using a combined genotype variant calling format (VCF) file merged from the genotype of HG01855 (see Additional file 4 for detail) and 1KG VCF file (containing the genotypes for all 2504 1KG samples, https://www.internationalgenome.org/category/vcf/). It showed again HG01855 of PRJEB31736 was grouped with AFR but not EAS samples (Fig. 7b), suggesting HG01855 of PRJEB31736 was truly mislabeled and Kssd-w/-rs is reliable for population-level clustering. Since PRJEB31736 is just the resequencing project of the 2504 1KG samples, the real label for HG01855 of PRJEB31736 must be one of the 2504 samples other than HG01855. Genotypes PCA showed HG01855 of PRJEB31736 was closest to HG01885 (but distant to HG01855) of 1KG (Fig. 7b), promoting HG01885 might be the real label for HG01855 of PRJEB31736. QTLtools mbv was then performed to confirm the real identity of HG01855 by matching the HG01855 bam file (generated by mapping all HG01855 runs to the chromosome 1 reference) to 1KG VCF file [17]. It showed again HG01855 of PRJEB31736 was best matched to HG01885 of 1KG, with genotype consistencies of 0.99 and 0.91 in heterozygous and homozygous sites, respectively (Fig. 7c). Inspired by QTLtools mbv, we created reference subtracted sketches for all 1KG samples (using chromosome 1 VCF file), and then computed the Jaccard and containment coefficients (Eqs. 2 and 3 in the “Methods” section) between HG01855 of PRJEB31736 and all 1KG samples. It showed again HG01885 was best matched, with Jaccard and containment coefficients of 0.0173 and 0.85, respectively (Fig. 7d). Moreover, HG01885 was clearly separated from other samples which were clumped together (Fig. 7c and d), which indicated that the matchings were significant. Therefore, the real label of sample HG01855 in PRJEB31736 should be HG01885—an AFR sample. The label HG01855 looks so similar with HG01885 (only different at the 6th character) that might have confused the data submitter and caused the mislabeling. A similar analysis on 19,326 human exome runs using Kssd-w/-rs alone found sample NA12275 of PRJNA59853 should be NA12775.

In the above experiment, we illustrated two ways of mislabeling detection—clustering based and matching based. Clustering-based way can only detect across-population mislabeling but need no reference datasets whereas matching-based way requires reference datasets (e.g., the 1KG VCF file in this experiment) but can both detect and correct mislabeling without the across-population limitation (e.g., both NA12275 and NA12775 are CEU samples). Kssd supports both of the two ways. Though the same analyses could also be done using convention methods like genotypes PCA and QTLtools, the computational expenses were enormously higher. Genotypes PCA and QTLtools sample matching took 1440 and 420 min respectively to test just one sample. Regardless of the raw datasets downloading, the main cost lay in bwa mapping and GATK [18] variant calling (only for genotypes PCA). In contrast, the main cost of Kssd lay in raw datasets sketching which took on average only 1.6 min per run for local datasets. In practice, Kssd sketching was piped from data streaming thus completed immediately after the datasets were fully fastq-dumped and there was almost no storage cost for the raw datasets. Once all the runs were sketched, the set operations and analyses on the sketches, including the reference subtraction, distance calculating, and sample matching, completed instantly (Fig. 7e).

To test if the reference subtraction step is essential, 160 runs (Additional file 3: Table S2) were randomly sampled and sketched, and pairwise distances were estimated using Mash (with sketch size of 500,000, minimum *k*-mer occurrence of 2) and Kssd-w/o-rs and Kssd-w/-rs (4096-fold dimensionality reduction, minimum *k*-mer occurrence of 2), respectively. MDS plot showed both Mash and Kssd-w/o-rs failed to cluster the runs (Fig. 7f and g), whereas Kssd-w/-rs unambiguously clustered the runs by population (Fig. 7h), which suggested that the reference subtraction essentially improved resemblance and containment measurements for population datasets.

## Discussion and conclusions

While we compare Kssd with other sketching techniques in this study, there is another class of sequence summarizing techniques named HyperLogLog, including HyperMinHash [19] and Dashing [20], which can also measure the distance for datasets using summaries. HyperLogLog do not fall into our category of sketching technique, as its summary saves no representative *k*-mers or hashes but a collection of estimators for the cardinality of *k*-mers set. Therefore, it cannot be applied to cases unitizing representative *k*-mers/hashes, e.g., sequences overlapping/mapping. HyperLogLog has its main superiority in summarizing speed, but slower in distance computing than Bindash and has no clear superiority in accuracy [20] and hence excluded for comparison in this study. There are also others theoretical developments on MinHash methods, e.g., SuperMinHash [21] and BagMinHash [22], but their practical purposes in biological datasets analysis are not clear yet.

Kssd uses a one-to-one function mapping from *k*-mer space to integer space, which is simpler than hash and has no collision and hence makes Kssd faster and more accurate than current sketching methods. More importantly, the one-to-one nature guarantees the security of set operations on sketches (Eqs. 7–10). Though a newly released MinHash library SourMash [23] also supports set operations on sketches, its security is not guaranteed due to the many-to-one nature of MinHash. Kssd support set operations of union, intersection, and subtraction. The reference subtraction operation not only reduces further the sketch size and hence save the computing cost, but also essentially improves the accuracy of distance estimation, hence for the first time enabling real-time and large-scale datasets clustering, mislabeling detection, and correction at population resolution. Union and intersection also have potential applications: for example, we could sketch all bacteria references and pool the sketches into a single one using union operation. The pooled sketch could be used as a contamination filter, and it could be subtracted from any given sketch, say a sketch of human WGS run, to filter out bacteria contaminations. We could create a pan-genome union sketch using 1000 genome project VCF file, and intersect it with sketch of an ancient human dataset to get a purified sketch for the dataset, so that we could cluster the purified sketch with sketches of 1000 genome project samples for fast ancestry test for the ancient human.

Kssd is highly scalable. To the best of our knowledge, the containment analyses on 1,019,179 bacteria runs is so far the largest analysis performed on a client-side machine (except those performed on the server-end machine of the database), and it created a combined Kssd sketch of only 6.7 Gb from the 1.4 Pb raw datasets. Though we have fastq-dumped almost all the bacteria datasets, it suffered significantly higher frequent fastq-dump failures on complicated datasets such as metagenomics and human WGS datasets, which prevented us from sketching the entire SRA database. However, for a local manager of NCBI or other large-scale databases, it is feasible to sketch all nucleotide datasets in the database. The superiority of Kssd in low-cost computing makes it possible to provide handy and real-time online analyses for much more simultaneous users hence may greatly fasten data-driven discoveries. It is also possible to store the combined Kssd sketch of all the nucleotide datasets from NCBI in a normal laptop or smartphone and use it in conjunction with a portable sequencing device like nanopore Minion, so that one can conduct real-time resemblance and containment analysis for sequencing data in areas where internet is inaccessible, e.g., fieldwork or remote area.

Though Kssd possess above-mentioned merits, there are still limitations: firstly, Kssd loses its efficiency when searching individual gene or small virus references contained in mixture of sequences since these sequences are too short to be dimensionality reduced further to an informative sketch [9]. And Kssd requires the mixture of sequences to be dimensionality reduced at the same folds with the references, so the mixture of sequences cannot be dimensionality reduced either. We suggest that the references should be at least 10,000 base pairs, so that the lowest-level sketches (with 16-fold dimensionality reduction) would still be informative enough for effective distance estimation. For single-gene searching, the tool BIGSI [24] would be a more suitable choice, and for numerous small virus genome searching, Mash screen would be better. Secondly, in the current version of Kssd implementation, the choices for the dimensionality reduction are restricted to 16^*z*^ fold (*z* takes integers), which prevents fine-tuning of the dimensionality reduction folds. Lastly, Kssd do not support amino acid sequences for current version.

One important future direction is developing long-reads overlapper and mapper based on the *k*-mer substring space sampling framework. Current long-read overlapper adopts the MinHash technique that always selects a fixed number of *k*-mers for a read [7], which may waste space or hurt sensitivity when input sequences vary greatly in lengths [8]. Kssd has the superiority in that it always selects *k*-mers with its number proportion to the length of the read. Moreover, Kssd sketch is reversible, meaning the selected *k*-mers could be recovered from the sketches, so there is no need to store the *k*-mers hence helps saving the memory cost. Another important direction is to estimate directly from the sketch the abundance of each reference contained. Though it is easy to tracking in sketch the *k*-mer occurrences from the mixture of sequences, the relationship between the *k*-mer occurrences and the abundance of each reference contained is complicated, since other genomes contained may also contribute to the *k*-mer occurrences thus confounding abundance estimation. However, the relationship is not totally chaotic, it is evidenced that there is clear average nucleotide identity (ANI) boundary (ANI = 95%) between two difference prokaryotic species [15], which means roughly 5% or larger genome regions are species-specific; thus, the *k*-mer occurrences of these regions are not or much less confounded by other species and probably reflect the truth abundance. Therefore, a robust inferring of species-specific *k*-mer occurrences would be the key for sketch-based abundance estimation. Though MinHash technique was introduced for biological sequences handling recently, it has long history of development that initially used for detecting similar web pages and clustering documents [5]. In contrast, Kssd was introduced first time in this study, but we speculate that a modified version of Kssd can perform tasks like documents clustering or similar web pages detection as well. After all, the documents, webpage content, and DNA are essentially all sequences but with a different alphabet set.

## Methods

### *k*-mer substring selection pattern and *k*-mer substring space sampling/shuffling and recoding

A *k*-mer substring selection pattern (kssp) *p* is a “01” string of length *k*, by which the letters at the 1s of the given *k*-mer (or *k*-mer set) *K* are concatenated, termed as the *p*-selected-*K*-substring(s) and denoted by *p*^1^(*K*), and the letters at 0 s are also concatenated, termed as *p*-unselected-*K*-substring(s) and denoted by *p*^0^(*K*). The weight of *p*, denoted by *w*, is the number of 1s in *p* (also the length of *p*^1^(*K*)). Given alphabet set *Σ* and weight *w*, the *k*-mer substring space ***S*** is the collection of all length *w* strings defined in *Σ* and thus has dimensionality |***S***| = |*Σ*|^*w*^ (we view each element in ***S*** as a dimension). ***S*** is first Fisher-Yates shuffled [25] and then partitioned into *N* subspaces of equal size, denoted by ***s***_***1***,_ ***s***_***2*,**_ *…*, ***s***_***N***_; for computational convenience, we choose *N* = |*Σ*|^*a*^, where *a* could be any positive integer ≤ *w*, so that |***s***| = | *Σ* |^*w-a*^ (***s*** ∈ {***s***_***1***,_ ***s***_***2*,**_ *…*, ***s***_***N***_}), and the dimensions of ***s*** could be represented by lexically ordered strings of length *w-a* (Fig. 2a).

Let *A* be a *k*-mer set; the subset *R*(*A*) = {*K*|*K* ∈ *A*, *p*^1^(*K*) ∈ ***s***} is mapped to sketch *S*(*A*) as follows: for each *k*-mer *K* ∈*R*(*A*), the *p*-selected-*K*-substring *p*^1^(*K*) is recoded by the lexical order of its dimension in the subspace ***s***, denoted by *s*(*p*^1^(*K*)), and then the *p*-unselected-*K*-substring *p*^0^(*K*) and the string *s*(*p*^1^(*K*)) are concatenated into the new string *p*^0^(*K*)*s*(*p*^1^(*K*)), which has length of *k*-*a* (Fig. 2b). This *k*-mer recoding scheme could be summarized by the one-to-one function *r*(*K*) = *p*^0^(*K*)*s*(*p*^1^(*K*)) mapping from *k*-mer to integers, hence avoiding hash collisions. The overall sketching process could be summarized by:

1 $$ S(A)=r\left(R(A)\right)=\left\{{p}^0(K)s\left({p}^1(K)\right)|K\in A,{p}^1(K)\in \boldsymbol{s}\right\} $$

### Kssd distance

The Jaccard and the containment coefficients for a *k*-mer set pair (*A*, *B*) are estimated by:

2 $$ \hat{J}\left(A,B\right)=\frac{\mid S(A)\cap S(B)\mid }{\mid S(A)\cup S(B)\mid } $$

and

3 $$ \hat{C}\left(A,B\right)=\frac{\mid S(A)\cap S(B)\mid }{\mathit{\min}\left(|S(A)|,|S(B)|\right)} $$

respectively. $\hat{J}$and $\hat{C}$could be further converted to Mash and Aaf distances by.

4 $$ \hat{D_m}=-\frac{1}{k}\mathit{\ln}\left(\frac{2\ \hat{J}}{1+\hat{J}}\right) $$

and

5 $$ \hat{D_a}=-\frac{1}{k}\mathit{\ln}\left(\hat{C}\right) $$

respectively; both the Mash and Aaf distances estimate the mutation distance between *A* and *B* [3, 13].

### Statistical properties of Kssd

Because of the nature of Fisher-Yates shuffle [25], subspace ***s*** and sketch *S*(*A*) are an unbiased sampling (without replacement) of space ***S*** and the recoded *k*-mer set *r*(*A*), respectively, and we have |*r*(*A*)| = |*A*|, since the *k*-mer recoding function *r* is injective. Thus, we have:

6 $$ E\left\{|S(A)|\right\}=\frac{1}{N}\mid r(A)\mid $$

namely, the expected rate of dimensionality reduction is $\frac{1}{N}$ for sketch *S*(*A*).

For two *k*-mer sets *A* and *B*, we have:

7 $$ {\displaystyle \begin{array}{c}S\left(A\cap B\right)=\left\{{p}^0(K)s\left({p}^1(K)\right)|K\in A\cap B,{p}^1(K)\in \boldsymbol{s}\right\}\\ {}=\left\{{p}^0(K)s\left({p}^1(K)\right)|\kern0.15em K\in A,{p}^1(K)\in \boldsymbol{s}\right\}\cap \left\{{p}^0(K)\ s\left({p}^1(K)\right)|K\in B,{p}^1(K)\in \boldsymbol{s}\right\}\\ {}=S(A)\cap S(B)\end{array}}\kern3em $$

and similarly, we have:

8 $$ S\left(A\cup B\right)=S(A)\cup S(B), $$

9 $$ S\left(A-B\right)=S(A)-S(B) $$

and

10 $$ S\left(A\cup \mathrm{B}-B\right)=S(A)\cup S(B)-S(B) $$

namely, the orders of Kssd sketching operation and set operations (e.g., union, intersection and subtraction) are interchangeable. It ensures the security and accuracy of set operations on sketches which equivalent to first do set operations on the raw datasets and then perform sketching on that resulting raw datasets. Since (*A* ∩ *B*) ∩(*A* ∪ *B* − *A* ∩ *B*) *= ∅*, we have *|S*(*A* ∩ *B*)*| +* |*S*(*A* ∪*B* − *A* ∩ *B*)*| = |S*(*A* ∪ *B)|*, where *|S*(*A* ∩ *B)|* and | *S*(*A* ∪*B* - *A* ∩ *B*)*|* are independent. Therefore, we have:

$$ {\displaystyle \begin{array}{c}E\left\{\frac{1}{\ \hat{J}}\ \right\}=E\left\{\frac{\mid S(A)\cup S(B)-S(A)\cap S(B)+S(A)\cap S(B)\mid }{\mid S(A)\cap S(B)\mid}\right\}\\ {}=E\left\{\frac{\mid S(A)\cup S(B)-S(A)\cap S(B)\mid }{\mid S(A)\cap S(B)\mid }+1\right\}\\ {}\begin{array}{c}=E\left\{\frac{\mid S\left(A\cup B-A\cap B\right)\mid }{\mid S\left(A\cap B\right)\mid}\right\}+1\ \left(\mathrm{by}\ \mathrm{interchangeability}\right)\ \\ {}=\frac{E\left\{|S\left(A\cup B-A\cap B\right)\ |\right\}\ }{E\left\{|S\left(A\cap B\right)|\right\}}+1\ \left(\mathrm{by}\ \mathrm{independency}\right)\\ {}\begin{array}{c}=\frac{\frac{1}{N}\mid A\cup B-A\cap B\mid }{\frac{1}{N}\mid A\cap B\mid }+1\ \left(\mathrm{by}\ \mathrm{Eq}.6\right)\\ {}=\frac{\mathbf{1}}{\boldsymbol{J}},\end{array}\end{array}\end{array}} $$

namely,

11 $$ E\left\{\hat{J}\right\}=J, $$

similarly, we have:

12 $$ E\left\{\hat{C}\right\}=C, $$

which means $\hat{J}$ and $\hat{C}$ are unbiased estimates of *J* and *C*, respectively. Both $\hat{J}$ and $\hat{C}$ are essentially sample proportions, thus have asymptotical gaussian distribution *N*($\hat{p}$, $\sqrt{\frac{\hat{p}\ \left(1-\hat{p}\right)}{n}}$), *np* > 10, where $\hat{p}$ = $\hat{J}$, *n* = |*S*(*A* ∪ *B*)| or $\hat{p}$ = $\hat{C}$, *n* = *min*(|*S*(*A*)|, |*S*(*B*)|) when *p* = *J* or *C*, respectively. Therefore, the population standard deviation is given by.

13 $$ sd\left(\hat{p}\right)=\sqrt{\frac{\hat{p}\ \left(1-\hat{p}\right)}{n}} $$

and the 95% confidence interval (CI) is [$\hat{p}$ − 1.96 $\sqrt{\frac{\hat{p}\ \left(1-\hat{p}\right)}{n}}$, $\hat{p}$ - 1.96 $\sqrt{\frac{\hat{p}\ \left(1-\hat{p}\right)}{n}}$]. The *P* value is defined by:

14 $$ P\  value=P\left(z\ge \frac{\hat{p}}{\sqrt{\frac{\hat{p}\left(1-\hat{p}\right)}{n}}}\right) $$

where the random variable *z* follows the standardized normal distribution. To account for multiple testing problem, *Q* value *Q* is calculated by multiplying the *P* value *P* by the total number of comparisons, for example, for *x* queries search against *y* references, *Q* = *Pxy*.

### *k*-mer set decomposition

By applying Eq. 1 with subspace ***s***_***1***_, ***s***_***2***_, ..., ***s***_***N***_ individually, *k*-mer set *A* can be decomposed into a collection of sketches *d*(*A*) = {*S*_*1*_(*A*), *S*_*2*_(*A*), ..., *S*_*N*_(*A*)}, respectively. Since {***s***_***1***_, ***s***_***2***_, ..., ***s***_***N***_} is a partition of *k*-mer substring space ***S***, we have $\bigcup \limits_{i=1}^N{\boldsymbol{s}}_{\boldsymbol{i}}$ = ***S*** and ***s***_***i***_ ∩ ***s***_***j***_ = *Ø*, for 1 ≤*i* ≤*N* and 1 ≤*j* ≤*N*, therefore,

15 $$ \bigcup \limits_{i=1}^N{S}_i(A)=\left\{{p}^0(K){s}_i\left({p}^1(K)\right)|K\in A,{p}^1(K)\in \boldsymbol{S}\right\}=r(A), $$

and

16 $$ {S}_i(A)\cap {S}_j(A)=\left\{{p}^0(K){s}_i\left({p}^1(K)\right)|\kern0.15em K\in A,{p}^1(K)\in {\boldsymbol{s}}_{\boldsymbol{i}}\right\}\cap \left\{{p}^0(K){s}_j\left({p}^1(K)\right)|K\in A,{p}^1(K)\in {\boldsymbol{s}}_{\boldsymbol{j}}\right\}=\varnothing, 1\le i\le \mathrm{N},1\le j\le \mathrm{N}\kern20em $$

Thus, we have:

17 $$ \left|A\right|=\left|\bigcup \limits_{i=1}^N{S}_i(A)\right|=\sum \limits_{i=1}^N\mid {S}_i(A)\mid . $$

The above conversion from *A* to *d*(*A*) is termed as *A* decomposition and sketches *S*_*1*_(*A*) ... *S*_*N*_(*A*) are termed as the components of *A*. Based on Eqs. 7, 8 and 17, the ground truth Jaccard coefficient *J* and containment coefficient *C* of two *k*-mer set*s A* and *B* could be recovered by:

18 $$ J=\frac{\mid A\cap B\mid }{\mid A\cup B\mid }=\frac{\sum \limits_{i=1}^N\mid {S}_i\left(A\cap B\right)\mid }{\sum \limits_{i=1}^N\mid {S}_i\left(A\cup B\right)\mid }=\frac{\sum \limits_{i=1}^N\mid {S}_i(A)\cap {S}_i(B)\mid }{\sum \limits_{i=1}^N\mid {S}_i(A)\cup {S}_i(B)\mid } $$

and

19 $$ C=\frac{\sum \limits_{i=1}^N\mid {S}_i(A)\cap {S}_i(B)\mid }{\min \left(\sum \limits_{i=1}^N|{S}_i(A)|,\sum \limits_{i=1}^N|{S}_i(B)|\right)}, $$

respectively. Equations 18–19 allow estimating the intersection and union of *A* and *B* component by component, where each component take only $\frac{\ 1\ }{N}$ memory of the *k*-mer set, which is particularly useful when ground truth *J* and *C* is needed in a memory-limited computer.

### *k*-mer length

The optimal *k*-mer length for alignment-free sequences comparisons is roughly (but not strictly) framed by the Eq. S2 in our previous work [26], namely:

20 $$ k={\log}_4\frac{3g}{2u}, $$

where *g* is the genome-size, and *u* is the allowed upper bound of the probability of random *k*-mer hitting ranging from 0.001 to 0.01 [26]. It yields *k* = 16 for bacterial or smaller genomes, *k* = 20 or 22 for metagenomics/mammals or larger genomes, and *k =* 18 for other genomes in between.

### Implementation

*k*-mer substring space sampling/shuffling is implemented by the subcommand shuffle, for example:

Kssd shuffle -k 9 -s 6 -l 3 -o out

This command will generate a file named “out.shuf” which keeps the shuffled *k*-mer substring space; this file would then be taken as input for sequences sketching or decomposition. To simplify kssp setting, kssd uses symmetric kssp “0{*x-y*}1{*y*}1{*y*}0{*x-y*},” where *x* and *y* are the half-length of *k*-mer and the half-length of *k*-mer substring (or kssp weight *w*), respectively, namely, *k* = 2*x* and *w =* 2*y*. 0{*x-y*} and 1{*y*} mean a 0s string of length *x-y* and 1s string of length *y*, respectively. Users need only set *x* (here by -k 9), *y* (here set by -s 6), and the dimensionality reduction level *z* (here set by -l 3; for decomposition purpose set -l 0). Referring to the number of partitions of *k*-mer substring space (or the dimensionality reduction folds) in the last section *N* = |*Σ*|^*a*^, where *a* = 2*z* here, so -l 3 set dimensionality reduction folds to 16^3^ for DNA sequence analysis. For the current version of kssd, *x*, *y*, and *z* should satisfy 12 > *x* > *y* > *z* + 2 > 1. *y* is suggest to greater than *z* + 2 to ensure the substring space after dimension-reduction is still large enough (greater than 16^2^) for robustness consideration.

To sketch/decompose reference sequences, just run:

Kssd dist -r <reference files dir> -L out.shuf -o <ref_outdir>

or

Kssd dist -r <reference files dir> -L 3 -k 9 -o <ref_outdir>

Either command will generate a database of reference-sketches in folder ref_outdir/ref.; the latter one actually combines the two commands below internally:

kssd shuffle -k 9 -s 6 -l 3 -o <ref_outdir>/default.shuf &&

kssd dist -r <reference files dir> -L ref_outdir/default.shuf -o <ref_outdir>

It simplifies the process at the cost of losing control for option “-s.”

To sketch/decompose query sequences, make sure using the same “.shuf” file with the references, and just run:

kssd dist -o <qry_outdir> -L out.shuf|<ref_outdir>/default.shuf <query files dir>

It will generate a combined queries sketch in folder qry_outdir/qry. Then, we can perform all queries versus all references comparisons by:

kssd dist -r <ref_outdir>/ref -o <outdir> <qry_outdir>/qry

To compare references to themselves, just run:

kssd dist -r <ref_outdir>/ref -o <outdir> <ref_outdir>/qry

Then, the distance will output to the “distance” file in the folder <outdir>.

For set operations, using:

kssd set -u <qry_outdir/qry> -o <union_outdir>

to create the union sketch in <union_outdir> from the combined queries sketch in <qry_outdir/qry>. Note the combined queries sketch is just a sketch combined from all queries sketches, the union operation deduplicate those integers duplicated in different queries;

and using:

kssd set -i <union_outdir> -o <intersect_outdir> <qry_outdir/qry>

to create the intersection sketch in <intersect_outdir> between the union sketch in <union_outdir> and the combined queries sketch in <qry_outdir/qry>;

and using:

kssd set -s <union_outdir> -o <subtract_outdir> <qry_outdir/qry>

to subtracts the union sketch in <union_outdir> from the combined queries sketch in <qry_outdir/qry> and creates the remainder sketch in <subtract_outdir>.

For large-scale remote datasets sketching, data streaming could be piped to Kssd sketching using:

kssd dist -L <default.shuf> -o <out_dir> --pipecmd “fastq-dump --skip-technical -Z” <Accession>

This command creates a sketch in <out_dir> for the remote run <Accession> directly without saving the run in local hence dramatically reduced the storage usage.

## Supplementary Information

**Additional file 1: Fig. S1.** Accuracies of kssd, mash and bindash on distant-related group.

**Additional file 2: Table S1.** 6164 Species Inconsistency Runs.

**Additional file 3: Table S2.** 160 randomly selected Runs.

**Additional file 4.** Supplementary Methods, the detailed workflow to reproduce the “Results”.

**Additional file 5.** Review history.

## Data Availability

The data accession numbers that support the findings of this study are available in Additional file 2: Table S1. All k-mer substring space file (.shuf file), sketch database, and the annotations of all analyzed datasets are available in https://drive.google.com/file/d/1VQOKGCXoSCmSesS-4Bn6SFJPO1YUY_b3/view?usp=sharing; https://drive.google.com/file/d/19zkKFalXXXdcMF8gFF-4DjxXrz1mOXDZ/view?usp=sharing. Kssd is freely available under an Apache License, Version 2.0 (https://github.com/yhg926/public_kssd) [10]; Kssd is also available at zenodo with DOI: 10.5281/zenodo.4438337 [11].
