# Supplementary material for: Kssd: sequence dimensionality reduction by k-mer substring space sampling enables real-time large-scale datasets analysis
Source: Genome Biol. 2021 Mar 16;22:84. doi: 10.1186/s13059-021-02303-4 (PMC7962209; doi:10.1186/s13059-021-02303-4)
Supplement: Supplementary file 1 — Additional file 1: Fig. S1. Accuracies of kssd, mash and bindash on distant-related group. [file 13059_2021_2303_MOESM1_ESM.pdf]

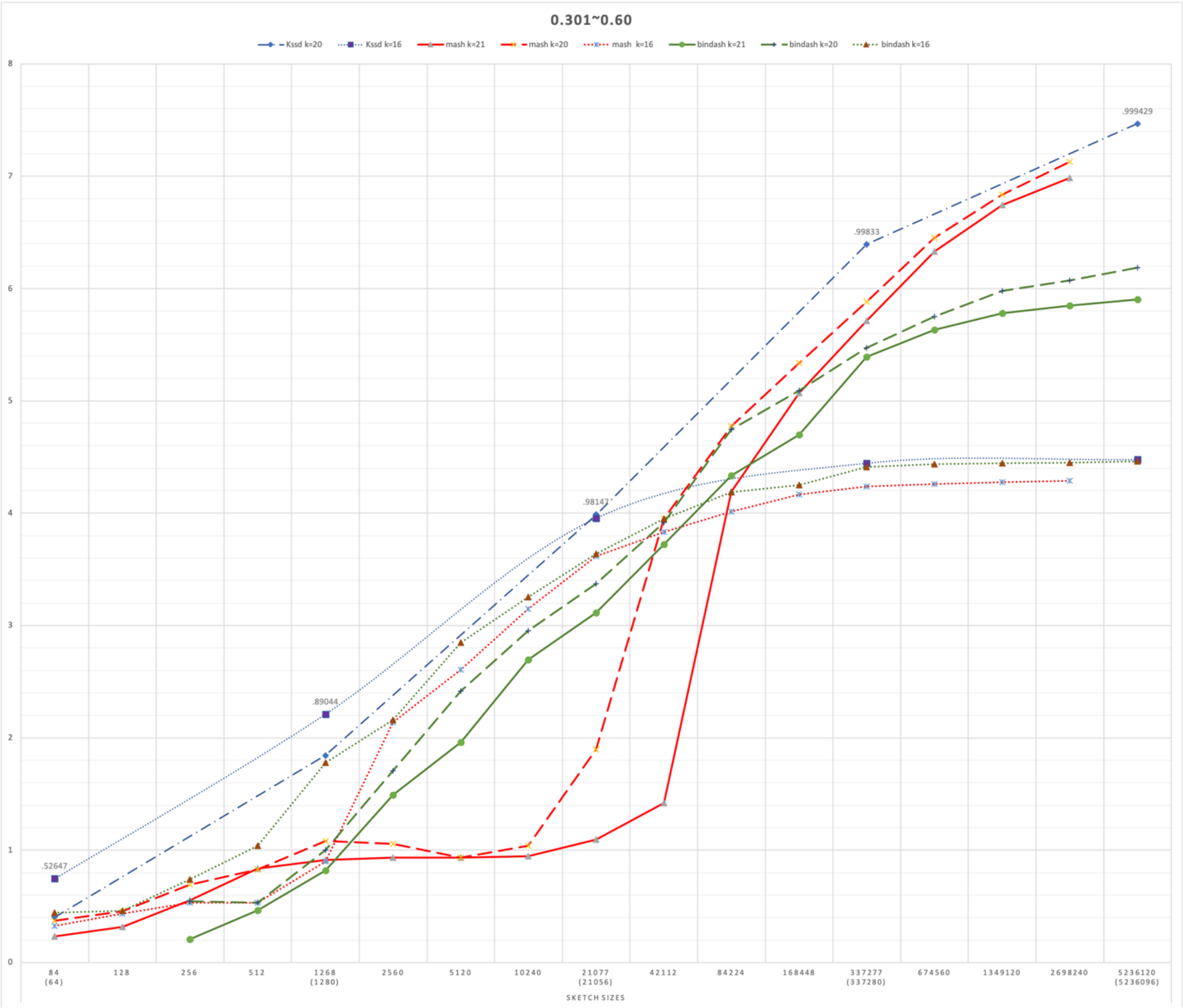

Fig. S1 Accuracies of kssd, mash and bindash on distant-related group. The distant-related group include 300 genomes in-silico evolved from a reference genome with 300 pre-defined mutation rates (ground truths) ranging from 0.301 to 0.60 with stepwise increasing of 0.001. The mutation rates were estimated by kssd, mash and bindash, respectively. Then Pearson correlation-coefficients *r* between the ground truth- and the estimated- mutation rates were calculated for the three methods with variated sketch-seizes (*x*-axis). *r* is scaled to $-\log(1 - r)$ for plotting clarity (*y*-axis). The decimal above the highest data point at a sketch-size is the maximal *r* value of all the three methods of all *k* settings with that sketch-size. The default *k*-mer lengths *k* for kssd, bindash and mash are 16, 21 and 21, respectively; to match *k*, we also run mash and bindash with *k* = 16 in addition to the default *k* settings, but kssd takes only even *k*, so we also run with *k* = 20 to vary *k*. Due to different sketching mechanism, mash and bindash take as parameter the sketch-size of continuous integers and multiples of 64,

respectively; but sketch-size is not a parameter of kssd and can only counted from the sketch file. To match sketch-sizes as closely as possible, we first sketched the reference using kssd with dimensionality-reduction levels n = {4, 3, 2, 1, 0} and obtained the sketch-sizes s_k = {84, 1268, 21077, 337277, 5236120}, respectively; we got the nearest multiples of 64 of s_k (the parenthesized values) and interpolated with their 2-, 4- and 8-fold sketch-sizes to obtain the sketch-sizes parameter s_b for bindash; and we merged s_k and the interpolated points of s_b to obtain the sketch-size parameter s_m for mash. Mash at sketch-size 5236120 is not shown due to the running error. Bindash with k=20, 21 at sketch-size 64 and 128 are not shown due to the estimates lacking of variation for correlation.
