# Supplementary material for: Kssd: sequence dimensionality reduction by k-mer substring space sampling enables real-time large-scale datasets analysis
Source: Genome Biol. 2021 Mar 16;22:84. doi: 10.1186/s13059-021-02303-4 (PMC7962209; doi:10.1186/s13059-021-02303-4)
Supplement: Supplementary file 4 — Additional file 4. Supplementary Methods, the detailed workflow to reproduce the “Results”. [file 13059_2021_2303_MOESM4_ESM.pdf]

Supplementary Methods

This document is the Supplementary Methods for reproducing the "Results" of the manuscript "Kssd: Sequence

Dimensionality-Reduction by K-mer Substring Space Sampling Enables Real-time Large-Scale Dataset Analysis".

For large-scale analysis, kssd will generates very large distance file and it take times to write large files.

To achive best performance, all our workflows were performed and tested on SSD disk. To reproduce, we also

recommend writing on SSD, it will be significantly faster then on spinning disk.

The full datasets including all sketches generated, the testing datasets, scripts, the shuffled k-mer substring space file (.shuf file)) used in this could be download from:

https://drive.google.com/file/d/19zkKFalXXXdcMF8gFF-4DjxXrz1mOXDZ/view?usp=sharing.

and

https://drive.google.com/file/d/1VQOKGCXoSCmSesS-4Bn6SFJPO1YUY_b3/view?usp=sharing

Accuracy of resemblance estimation

By Huiguang Yi

Email: yhg926@gmail.com

2019-08-08

---

0. Introduction

This is the workflow used to generate this part of Results: Accuracy of resemblance estimation (Figure 1)

Softwares versions in this experiment: kssd version 1.0, bindash version 0.2.1 and Mash version 2.0.

This experiment performed under linux system, 32G and 12-cores machine.

1. Inventary

1.1 Testing datasets

The folder `dist1_30` includes AE016877.fasta and its 300 mutants with mutation rates range from 0.001 ~ 0.300; The folder `dist31_60` inclu AE016877.fasta and its 300 mutants with mutation rates range from 0.301 ~ 0.600. The mutation rate of each mutant showed as the decimal b 'AE016877.' and '.fasta', for example: AE016877.0.128.fasta has a mutation rate of 0.128 from AE016877.fasta. These mutants were generated u script `fasta_mut.pl`.

1.2 kssd_shuf_files

The .shuf files used by kssd for sketching.

1.3 Sketch-sizes file

bindash.sizes.txt and mash.size.txt listed the sketch-sizes for bindash and mash, respectively.

1.4 resemblance_accuracy.xlsx

This is the same data of Figure 1. showed in a table.

2. Methods

2.1 kssd

2.1.1 sketching

We first sketched `dist1_30` and `dist31_60` by dimensionality-reduction levels l = {4, 3, 2, 1, 0} and k = {8,10}, where k is half length of k-length in kssd (k=16,20). The folder `kssd_shuf_files` contains all the '.shuf' files used in the kssd resemblance estimation (manuscript Figure except l = 0, which do not need a '.shuf' file (no dimensionality-reduction); File names follow this format: L l K k _ $group$ .shuf, where l , k ar $group$ are the arguments of dimensionality-reduction level, k-mer length and mutants groups, respectively. Here is an example for sketching:

```
kssd dist -L kssd_shuf_files/L3K8_dist1_30.shuf -r dist1_30 -o L3K8_dist1_30
```

You can sketching using other .shuf files to get sketches with variated parameters.

For k =10 && l < 2, the k-mer substring space overflows this kssd implementation; then kssd need to be re-compiled; go to the kssd installa directory and type:

```
make strange
```

and run using the 'strange' kssd implementation, for example:

```
kssd dist -L 0 -k 10 -r dist1_30 -o L0K10_dist1_30
```

but do not use this 'strange' kssd implementation unless necessary, it is inefficient.

2.1.2 Distance estimation

The pair-wise distance file `L3K8_dist1_30/distance` were generated as follow (use `L3K8_dist1_30` as an example here):

```
kssd dist -r L3K8_dist1_30/ref -o L3K8_dist1_30 L3K8_dist1_30/qry
```

distance file for all other sketches were generated similarly.

2.1.3 Correlation with ground truth mutation rates

Use `L3K8_dist1_30` as an example:

```
cd L3K8_dist1_30 &&
awk '$1 ~ /dist1_30\/AE016877.fasta/ && $2 !~ /dist1_30\/AE016877.fasta/' ./distance.out|cut -f2,5|grep -v 'inf'|sed 's/\
(dist1_30\/AE016877\.\|\.fasta\)//g'|sort -k1 -g|Rscript -e ' v<-read.table(file("stdin"),sep="\t");cor(v[,1],v[,2])'

[1] 0.9958683
```

all other correlation-coefficients were obtained similarly.

2.2 bindash

2.2.1 Sketching

```
for i in `cat bindash.size.txt`; do bindash sketch --kmerlen=16 --sketchsize64="$i" --outfname=bindash.dist1_30k16s"$i".sketc
listfname=bindash_dist1_30.list --nthreads=12; done
```

`bindash.size.txt` includes all sketch-sizes set for the option --sketchsize64; `bindash_dist1_30.list` contain fullpaths for sequences in `di`
This is an example sketching using bindash with k=16 on `dist1_30`; You can perform sketching similarly using k = {20,21}, and on folder `dis`

2.2.1 Distance estimation

```
mkdir bindash_out &&
mv *.sketch bindash_out/ &&
cd bindash_out/ &&
for i in `find ./*.sketch`; do bindash dist $i $i >$i.distance;done
```

2.2.2 Correlation with ground truth mutation rates

```
 for i in `find ./bindash.dist1_30k20s*.sketch.distance`; do j=`awk '$1~/\/AE016877.fasta/ && $2!~/\/AE016877.fasta/' "$i" |c
f2,3|sed 's/\(\.\.\/dist1_30\/AE016877\.\|.fasta\)//g' | sort -k1 -g |Rscript -e ' v<-
read.table(file("stdin"),sep="\t");cor(v[,1],v[,2])'`;echo $i" "$j; done|sed 's/\(\.\/bindash\.dist1_30k20s\|\.sketch\.distar
[1\]\)//g' |sort -k1 -g
```

This gave Correlation-coefficients of bindash distance (of all sketch size) with ground truth, using k = 20, on folder `dist1_30`. You can do si
using k ={16,21}, and on folder `dist31_60`.

2.3 Mash

2.3.1 Sketching

```
for j in `cat mash.size.txt|grep -v '5236120'`; do mash sketch -k 20 -s $j ../dist31_60/*.fasta -o mash_k20s"$j"d31_60 -p 12;
```

This is an example for mash sketching using `dist31_60`, k=20 and all sketch-sizes in mash.size.txt except '5236120'; sketch-size '5236120' c
running error in my machine, but you can try it on your machine. And you can perform sketching similarly using k = {16,21}, and on folder `dis`

2.3.2 Distance estimation

```
mkdir mash_out &&
mv *.msh mash_out/ &&
cd mash_out &&
for i in `find ./*.msh`; do mash dist $i $i > $i.distance;done
```

2.3.3 Correlation with ground truth mutation rates

```
 for i in `find *.distance|grep -v '5236120'`; do j=`awk '$1 ~ /\/AE016877.fasta/ && $2 !~ /\/AE016877.fasta/' $i | cut -f2,3
sed 's/\(\.\.\/dist.*AE016877\.\|\.fasta\)//g' |sort -k1 -g|Rscript -e ' v<-read.table(file("stdin"),sep="\t");cor(v[,1],v[,2
`;echo $i" "$j;done |grep 'd31_60'|grep 'mash_k21s'|sed 's/\(mash_k21s\|d31_60\.msh\.distance \[1\]\)//g' |sort -k1 -g
```

This gave Correlation-coefficients of mash distance (of all sketch-sizes except 5236120) with ground truth, using k = 21, on folder `dist31_6`
can do similarly using k ={16,20}, and on folder `dist1_30`.

Accuracy of containment estimation

By Huiguang Yi

Email: yhg926@gmail.com

2019-08-08

0. Introduction

This is the workflow used to generate this part of Results: Accuracy of containment estimation (Figure 2)

Softwares versions in this experiment: kssd version 1.0, Mash version 2.0.

This experiment performed under linux system, 32G and 12-cores machine.

1. Inventory

1.1 64constitutes

This folder containts the 64 known constitute-genomes of the skakya dataset.

1.2 assembly_summary.txt

This is the summary of the 138,743 reference-genomes, you can download .fna sequences follow the ftp links (20th Column).

1.3 L3K10.shuf

This is the `.shuf` file for this expriment, including sketching of the 138,743 reference, the 64 constitute-genomes and the skakya dataset(bot and simulated).

1.4 Sketches

This folder contain three folders `ref`, `64constitute_qry` and `shakya_qry`, they are the sketches of the 138,743 references, 64constitutes an datasets,respectivly. You can use these sketches to compute distances directly (skip to section 2.4), but replace the parameters' names respe

1.5 SourceData

This folder contains two files SRR606249.kssd.csv and simshakya.kssd.csv, they are the source .csv files of minimum reference-to-constitutes containement-measurements for real and simulated shakaya dataset, used to generate Figure 2.

2. Methods

You can skip to 2.4 if you do not need sketch the sequences (We have already provided all the sketches in folder `Sketches`). Otherwise, you c these steps:

2.1 Download all references and skakya dataset

You can download them yourself or using the below bash scripts:

```
#for references
for i in `cut -f20 assembly_summary.txt|tail -n +3`; do { j=`basename $i`; wget -c $i/"$j"_genomic.fna.gz -O
./ncbi_bacteria_gnome/"$j".gz ; wget -c $i/md5checksums.txt -O ./ncbi_bacteria_gnome/"$j".mds;}& done
#re-download for failed files
for i in `cut -f20 assembly_summary.txt|tail -n +3`; do { j=`basename $i`; [ -s ./ncbi_bacteria_gnome/"$j".gz ] || wget -c
$i/"$j"_genomic.fna.gz -O ./ncbi_bacteria_gnome/"$j".gz ; wget -c $i/md5checksums.txt -O ./ncbi_bacteria_gnome/"$j".mds;}& do

#for the real skakya datasets
fastq-dump -A SRR606249
```

2.2 Generated the simulated skakya dataset

The steps strictly follow the mash screen paper of Odove et. al.(2019)

```
cat 64constitutes/*.fasta > combined.shakya.fasta;

art_illumina -f 50 -l 100 -ss HS20 -i combined.shakya.fasta -o sim.combined.shakya
```

2.3 Sketch sequences

2.3.1 Sketch references

```
/usr/bin/time -v kssd dist -L L3K10.shuf -r ./ncbi_bacteria_gnome -o L3K10_ncbi_bacteria_gnome
```

2.3.2 Sketch 64 constitute-genomes

```
kssd dist -L L3K10.shuf -o L3k10_64constitutes ./64constitutes
```

2.3.3 Sketch the real and the simulated shakya dataset

```
mkdir shakya_sim_real_fastq &&
mv SRR606249.fastq sim.combined.shakya.fastq shakya_sim_real_fastq/
/usr/bin/time -v kssd dist -L L3K10.shuf -o L3k10_shakyafq shakya_sim_real_fastq
```

2.4 Kssd Distance estimation

2.4.1 64 constitute-genomes to reference distances

```
kssd dist -r L3K10_ncbi_bacteria_gnome/ref -o constitutes2ref L3k10_64constitutes/qry
#Or if you use Sketches folder
cd Sketches &&
kssd dist -r ref/ -o ../constitutes2ref 64constitute_qry &&
cd ../
```

2.4.2 Shakya to reference distances

```
kssd dist -r L3K10_ncbi_bacteria_gnome/ref -o shakya2ref L3k10_shakyafq/qry
#Or if you use Sketches folder
cd Sketches &&
kssd dist -r ref/ -o ../shakya2ref shakya_qry &&
cd ../
```

2.5 Plot containment-measurements against the minimum reference-to-constitutes distances

2.5.1 Get the minimum reference-to-constitutes distances

```
tail -n+2 constitutes2ref/distance.out |cut -f2,5 |sort -k1 -k2 |awk 'NR % 64 == 1' >best_hit_nbci130k_in_64_shakya.out
```

2.5.2 Intergrate containement-measurements with the minimum reference-to-constitutes distances && Plot && correlation

For real shakya dataset:

```
awk '$1 ~ /SRR606249/' shakya2ref/distance.out|cut -f2,7|sort -k1 >nbci130k_vs_SRR606249.out
paste best_hit_nbci130k_in_64_shakya.out nbci130k_vs_SRR606249.out|cut -f2,4|grep -v 'inf'|gnuplot -persist -e "plot '< cat'
paste best_hit_nbci130k_in_64_shakya.out nbci130k_vs_SRR606249.out|cut -f2,4|grep -v 'inf'|awk '$1 < 0.15'|Rscript -e ' v<-
read.table(file("stdin"),sep="\t");cor(v[,1],v[,2])'
```

For simulated shakya dataset:

```
awk '$1 ~ /sim.combined.shakya/' shakya2ref/distance.out |cut -f2,7|sort -k1 >nbci130k_vs_simshakya.out
paste best_hit_nbci130k_in_64_shakya.out nbci130k_vs_simshakya.out|cut -f2,4|grep -v 'inf'|gnuplot -persist -e "plot '< cat'
paste best_hit_nbci130k_in_64_shakya.out nbci130k_vs_simshakya.out|cut -f2,4|grep -v 'inf'|awk '$1 < 0.15'|Rscript -e ' v<-
read.table(file("stdin"),sep="\t");cor(v[,1],v[,2])'
```

2.5 Mash screen Distance estimation

Please refer to the mash screen paper of Odove et. al.(2019)

2.6 Containment analysis on 65265 Metagenomics

```
# download sketches using this link:
https://drive.google.com/file/d/1VQOKGCXoSCmSesS-4Bn6SFJPO1YUY_b3/view?usp=sharing

tar -xvf FileSL1_combined_metagenomics_65265_succed_sketches.tar.gz

# containment analysis

kssd dist -r Sketches/ref -o outdir FileSL1_combined_metagenomics_65265_succed_sketches
```

All bacteria WGS runs analysis

By Huiguang Yi

Email: yhg926@gmail.com

2020-05-24

---

0. Introduction

This is the workflow used to generate this part of Results: Optimal references and species inconsistency detection for all bacteria WGS datase

Softwares versions in this experiment: kssd version 1.1.

This experiment performed under linux system, 32G and 12-cores machine with SSD.

1. Inventory

1.1 Accessions File

- AllBacteriaRun_accession.txt
- 6164SpeciesInconsistencyRun.txt

1.2 Optimal references for all Bacteria Runs

- manuscript_1019179x138743_M1N10.optimal_references.txt

1.3 Pre-shuffled and sampled *k*-mer subtring space File

- L3K10.shuf

1.4 Sketches

- Prokayotic138743Refseq_indexedSketch
- 1M_bacteria_combined_sketches

1.5 taxid file for all runs and references

- species.taxid.1M_bacteria_ac.csv
- 130k_reference.taxid

1.6 perl scripts

- find_mis-track.pl
- distance_add_taxid.pl
- select_real_mistract.pl

2. Methods

You can skip to 2.2 if you do not need sketching the sequences (We have already provided all the sketches).

2.1 Sketching all Bactria Accessions from data streaming

```
mkdir -p $HOME/.ncbi
echo '/repository/user/main/public/root = "/tmp"' > $HOME/.ncbi/user-settings.mkfg
outdir="$(pwd)/allbacrun"
mkdir -p $outdir;
acln="$(pwd)/AllBacteriaRun_accession.txt"

for i in `cat $acln`;
  do rm /tmp/sra/* ; prefetch $i && kssd dist -L L3K10.shuf -n 2 -o $outdir/$i --pipecmd "fastq-dump --skip-technical --split
-Z" /tmp/sra/$i.sra ;
done

# Combine sketches
# if * matches exceed your command line arguments limitation,
#split sketches in batches, and combined by batch, and combined all batch sketches in final
kssd dist -o 1M_bacteria_combined_sketches $outdir/*/qry
```

2.2 Prioritizing 10 references for each runs

```
# Caculates containment Measurements
kssd dist -r Prokayotic138743Refseq_indexedSketch -O0 -M1 -N10 1M_bacteria_combined_sketches
# trim distance.out
sed 's/\/.*.sra\|..\/ncbi_bacteria_gnome201801230\/\|.fna.gz//g' ./distance.out |cut -f1,2,4 >
manuscript_1019179x138743_M1N10.optimal_references.txt
```

2.3 Species inconsisteny analysis

```
# compute all vs. all distance and print distances < 0.05
 kssd dist -r Prokayotic138743Refseq_indexedSketch -O0 -M1 -D 0.05 -N1024 1M_bacteria_combined_sketches

# identify Species inconsisteny runs
perl find_mis-track.pl 130k_reference.taxid species.taxid.1M_bacteria_ac.csv distance.out 0.05 > species.mistract.distance.ou
2>/dev/null

perl distance_add_taxid.pl 130k_reference.taxid species.taxid.1M_bacteria_ac.csv distance.out 0.05 2>/dev/null | perl
select_real_mistract.pl species.mistract.distance.out - > select.species.mistract.distance.out
```

1000 genome project datasets analysis

By Huiguang Yi

Email: yhg926@gmail.com

2020-05-24

---

0. Introduction

This is the workflow used to generate this part of Results: Population datasets clustering and mislabeling detection using kssd reference subtr
(Figure 5)

Softwares versions in this experiment: kssd version 1.1.

This experiment performed under linux system, 32G and 12-cores machine with SSD.

1. Inventory

1.1 Accessions File

- 1730_1kgPRJEB31736_accesssion.txt
- slct160.accesssion.txt
- 19326_1kgExome_accesssion.txt

1.2 Pre-shuffled and sampled *k*-mer subtring space File

- L3K10.shuf

1.3 Sketches

- hg38Ref_sketch
- VCF1kg2504RefSubtract_sketch
- 1730_1kgPRJEB31736RefSubtract_sketch
- exome19326_1kgRefSubtract_sketch

1.4 Population information file for all runs

- runid2pop.txt

1.5 Perl script

vcf2fasta.pl

2. Methods

You can skip to 2.3 if you do not need sketching the sequences and substracing refrence (We have already provided all the sketches). Otherwi can follow these steps:

2.1 Create Sketches

2.1.1 Sketching human reference

```
#Download human reference fasta file by your own.
#suppose your reference filename is hg38.fa, then sketch it by:
kssd dist -L L3K10.shuf -o hg38Ref_sketch $(pwd)/hg38.fa
# if you want to use it for subtraction operation, run:
kssd set -u -o hg38Ref_sketch hg38Ref_sketch/qry
```

2.1.2 Sketching VCF file and reference

```
#download 1KGP VCF file and reference (only need one chromosame)
 wget -c
http://ftp.1000genomes.ebi.ac.uk/vol1/ftp/release/20130502/ALL.chr1.phase3_shapeit2_mvncall_integrated_v5a.20130502.genotypes
gz;
 wget -c ftp://ftp.1000genomes.ebi.ac.uk//vol1/ftp/technical/reference/phase2_reference_assembly_sequence/hs37d5.fa.gz

#extract fasta file from VCF (chromosame 1 here)
 for i in `seq 1 50 2504`; do { perl vcf2fasta.pl chr1.hs37d5.fa
ALL.chr1.phase3_shapeit2_mvncall_integrated_v5a.20130502.genotypes.vcf $i 50 ;}& done

#collect all fasta in folder vcf_fas and sketching
 kssd dist -L L3K10.shuf -o chr1_vcf_fas_sketch vcf_fas

#sketching reference (chr1) and union it for subtraction
 kssd dist -L L3K10.shuf -o chr1_hs37d chr1.hs37d5.fa
 kssd set -u -o chr1_hs37d chr1_hs37d/qry/
```

2.1.3 Sketching all Accessions from data streaming

```
# Using 160 selected runs as example
mkdir -p $HOME/.ncbi
echo '/repository/user/main/public/root = "/tmp"' > $HOME/.ncbi/user-settings.mkfg

outdir="$(pwd)/slct160"
mkdir -p $outdir;
acln="$(pwd)/slct160.accesssion.txt"

for i in `cat $acln`;
  do rm /tmp/sra/* ; prefetch $i && kssd dist -L L3K10.shuf -n 2 -o $outdir/$i --pipecmd "fastq-dump --skip-technical --split
-Z" /tmp/sra/$i.sra ;
done

# Combine sketches
kssd dist -o ./slct160_sketch $outdir/*/qry
```

2.2 Reference subtraction

```
#for 1KGP VCF
kssd set -s chr1_hs37d -o VCF1kg2504RefSubtract_sketch chr1_vcf_fas_sketch/qry
# for other datasets
kssd set -s hg38Ref_sketch -o slct160_RefSubtract_sketch slct160_sketch/qry
...
```

2.3 Distance estimations using refrence subtracted sketch

```
# Use 1730_1kgPRJEB31736RefSubtract_sketch as an example
# user can replace with VCF1kg2504RefSubtract_sketch, slct160_RefSubtract_sketch and exome19326_1kgRefSubtract_sketch for ana
on corresponding datasets

#index sketch if want use it as a database.
kssd dist 1730_1kgPRJEB31736RefSubtract_sketch
#this command generate indexed sketch with default name 'ref'

# Using resemblance-measurements (-M0 in kssd v1.1) for clustering purpose
kssd dist -r ref -M0 -O0 1730_1kgPRJEB31736RefSubtract_sketch
#this command generated pairwise distance in file with default name "distance.out".

# trim "distance.out"
sed -i 's/\/[^\t]*//g' ./distance.out
```

2.4 MDS plot in R

```
library('rgl')
dist<-read.table("./distance.out",header=T)
nameVals <- sort(unique(unlist(dist[1:2])))
myMat <- matrix(0, length(nameVals), length(nameVals), dimnames = list(nameVals, nameVals))
myMat[as.matrix(dist[c("Qry", "Ref")])] <- dist[["MashD"]]
fit <- cmdscale(myMat,eig=TRUE, k=3)
x <- fit$points[,1]
y <- fit$points[,2]
cl<-read.table("./runid2pop",row.names = 1)
cl<-cl[names(x),]
Ncl<-length(levels(cl[,2]))
clr=rainbow(Ncl)[cl[,2]]
options(rgl.printRglwidget = TRUE)
plot(x,y, xlab="MDS1", ylab="MDS2",main="Runs Clustering (Reference Subtracted)", col = clr)
legend("topright", inset=.02, title="Super Population",
       as.character(unique(cl[,2])), fill= unique(clr), horiz=F, cex=0.8)
```

2.5 Sample Matching using 1KG VCF sketch

```
# Use 1730_1kgPRJEB31736RefSubtract_sketch as an example
# user can replace with exome19326_1kgRefSubtract_sketch for analysis on exome datasets

# Index VCF sketch
 kssd dist VCF1kg2504RefSubtract_sketch

# Resemblance (-M0) and Containment(-M1) distance
 kssd dist -r ref -M0 -O0 1730_1kgPRJEB31736RefSubtract_sketch && mv ./distance.out M0.distance
 kssd dist -r ref -M1 -O0 1730_1kgPRJEB31736RefSubtract_sketch && mv ./distance.out M1.distance

# for a given run, say ERR3576659
 grep 'ERR3576659' M0.distance >M0.ERR3576659
 grep 'ERR3576659' M1.distance >M1.ERR3576659
 paste M0.ERR3576659 M1.ERR3576659|cut -f2,4,9 |sed 's/vcf_fas\/\|.fas//g' > M0M1.ERR3576659_match_vcf

# plot in R
t<-read.table("M0M1.ERR3576659_match_vcf",header=T,row.names=1)
plot(t,main="ERR3576659 Kssd Samples Matching")

# for many sample analysis, you can write batch script finding inconsistent matched sample by your own
```
