# Supplementary material for: Kssd: sequence dimensionality reduction by k-mer substring space sampling enables real-time large-scale datasets analysis
Source: Genome Biol. 2021 Mar 16;22:84. doi: 10.1186/s13059-021-02303-4 (PMC7962209; doi:10.1186/s13059-021-02303-4)
Supplement: Supplementary file 5 — Additional file 5. Review history. [file 13059_2021_2303_MOESM5_ESM.docx]

Review History

**First round of review**

**Reviewer 1**

**Were you able to assess all statistics in the manuscript, including the appropriateness of statistical tests used?**

No, I do not feel adequately qualified to assess the statistics.

**Were you able to directly test the software?**

No

**Comments to author:**

The authors developed a sketching technique to calculate the resemblance (similarity) and containment of two DNA sequences. This can be done by generating the set of all k-mers (substrings of length k) from each sequence, and then computing the Jaccard (resemblance) and containment coefficients of the two sets. The authors improve the performance of this by creating sketches containing a much smaller number of recoded k-mers, and running the same set operations on these sketches. They do this by first selecting an unbiased sample s of the k-mer space S (all possible sequences of length k). The sketch of a sequence contains only the k-mers of the sequence that are in s. The authors tested dimension reductions up to where s is 16^-4 the size of S (keeping about 1/65536 of the k-mers). The recoding step reduces the length of the k-mers by leveraging the fact that they are all in s. They demonstrated several applications for their algorithm, such as detecting incorrectly labeled samples, in large datasets. Although computation on the sketches is orders of magnitude faster than other algorithms, when including the time to generate the sketches, the overall approach was 12-30 times faster and about 2 times more accurate. Though, subsequent analyses are much faster since the sketches have already been calculated.

Minor Comments:

1) The authors write that the k-mer substring selection pattern (e.g., "000010010000") is "pre-determined" for 12-mer analysis. How was this selected? What weight w should be used? What patterns should be used for k-mers and substrings of different lengths? Why not place the 1's at the end so that it is easier to construct p0(K) and p1(K)?

2) The notation seems to change throughout the manuscript, which makes it difficult to read. For example, the Kssd program has an input variable -s, which is the half-length of the k-mer substring. Line 419 calls this "m". Though, I think this is the same as "w" on line 349. On line 352, "m" is related to the dimension reduction and used to determine the number of partitions, N=4^m. The Kssd program has another variable -l, which is the rate of dimensionality reduction. However, instead of it being a power of 4, on line 421 it is a power of 16. Does this mean that N=4^(2*l)? In the Figure 1 caption, they use lower-case "n" for the dimensionality-reduction levels (0 to 4), which I assume means N=16^n. Equation 20 also uses "m", but for something completely different.

3) The algorithm includes both dimension reduction and recoding. How much of the performance gain is due to each part? Is the performance benefit of recoding greater than the time needed to recode?

**Reviewer 2**

**Were you able to assess all statistics in the manuscript, including the appropriateness of statistical tests used?**

There are no statistics in the manuscript.

**Were you able to directly test the software?**

No

**Comments to author:**

In the manuscript under consideration, the authors presented Kssd, a novel method that compares two set of genomes using a novel design of sketch strategy. It is actually quite difficult to read in terms of both methods and results. I have read it carefully twice and finally figured out the idea behind the methods. I feel the idea is inspiring and deserves a publication. However, I still have some reservations on some of the main claims of the results and have some suggestions for authors to improve the presentation (assuming my understanding of their messages is correct). I list the items below for the authors' consideration:

Claim that need to be strengthened:

First, there is a claim that Kssd uses 5 magnitudes lower storage in Abstract. However, it appears they used the size of the sketch set of Kssd to compare the size of raw data used by Mash. This is an unfair comparison. To use Kssd, one doesn't need to download raw data at all?

Second, there are two cool results from the real data analyses: the identification of incorrectly registered species in metagenomic data, and the incorrectly labeled human samples. I am curious whether this is the advantage of Kssd? It appears to me that other methods may also identify such mistakes using contamination analysis?

Presentation:

First of all, the inspiring idea that convinced me this is a great piece of work, depicted in Figure 6, may be better presented and presented upfront. I feel that the authors might explain the idea in Introduction and describe the algorithm in the first section of Results. They may want to move Figure 6 to Figure 1 so that the readers get their idea upfront.

Second, the motivation of the mathematical proofs in the Method section are not clear. What are their roles in the method development?

Third, the claim of identifying incorrect labelled samples are unclear for me. If I understood the results correctly, the authors have aligned the reads files to the VCF-file supported reference genome and identified inconsistency? So, the reads files are still the standard and the VCF files are mislabeled? Please clarify this result.

Fourth, there is a striking claim that the speed is 2500 times faster than state-of-the-art. However, this is not consistent with the data shown in Figure 3, the main display on CPU time. I carefully inspected the numbers and understood that Figure 3 is depicting something else different to the numbers they claimed in the abstract. I feel the authors should use a main display for their main claim in the abstract, instead of displaying other different calculations.

Fifth, there is a claim that "There is no need for Kssd to sketch the raw datasets again for the containment-analysis for new references". I can't understand why this is the case. When new references are added, we still need to generate the sub k-mer space for the new reference?

Sixth, it is claimed that the method is hash-free. I feel this is not necessary (if not incorrect). Kssd still uses hash however choose to hash a subset of the full k-mer space instead of a subset of the genomic sequences (that is species-specific). This is an excellent idea but why calling if hash-free? I have no problem if the authors really want to call it that way: but it adds a bit difficulty to understand what exactly the algorithm is.

Finally, why use line-chart in Figure 4? If the species depicted are different and we don't really expect to learn a trend, should we just use a bar-chart? Also, my understanding is that this figure plots the selected samples with incorrect registered references. If so, why are there a couple of outliers, SRR6045040 and ERR2789389)? Did I misunderstand your results?

**Reviewer 3**

**Were you able to assess all statistics in the manuscript, including the appropriateness of statistical tests used?**

Yes, and I have assessed the statistics in my report.

**Were you able to directly test the methods?**

No

**Comments to author:**

Yi et al. present a new, intuitive algorithm for representing a genome or genomic sequence data set in a highly compressed format (a "sketch") such that whole-genome comparisons can be performed very fast. These comparisons include measuring overall similarity of two genomes and detecting if a genome is "contained" in a metagenome. The authors demonstrate that the new tool (called kssd) is much faster and/or more storage-efficient than state-of-the-art in certain situations.

An impressive analysis is the detection of "species inconsistency", where a bacterial WGS data set is examined for containment of every bacterial reference genome and the best contained genome is found to be different from the species that the original data set was registered as. What is impressive is that over a million data sets are analyzed for containment of all prokaryotic genomes, resulting in over 100 billion containment coefficients, in only ~3 hours on a common 12 CPU machine. The species inconsistencies discovered are further confirmed as being real, although they are relatively rare (only ~6000 out of the ~1 M data sets, i.e., ~0.6%).

General comment: The authors present various evaluations and comparisons, and each highlights some positive aspects of the new tool, but also raises some questions about practical implications of those advantages. (See specific comments #2-4 below.) Perhaps the authors could highlight the most important application among these that they believe should convince the reader to adopt the new tool in place of the best alternatives. Since the main advantage overall is increased speed and/or decreased storage, the authors should try to argue that some task that *users will commonly want to perform* using sketches is *impractical currently* (e.g., will take days or months) but *can be made practical using kssd*. Without such a blunt argument, the merit of improved speed/storage is harder to appreciate.

Specific comments:

1)	To someone not familiar with sketching tools, the introduction does a poor job of explaining what exactly the goal is. Existing sketching techniques are introduced and critiqued (e.g., lines 65-75) but it is hard to appreciate those criticisms if one doesn't know exactly which biological tasks will utilize the sketching and how those biological tasks are impacted by the supposed weaknesses.

2)	Figure 1 is meant to show that mutation rates estimated using kssd are better than those estimated using existing methods mash and bindash. However, it is not clear what is the practical value of an improvement in the fourth significant digit of the correlation coefficient between predicted and "real" mutation rates.

3)	Figure 2 is meant to compare kssd and an existing method "mash screen" for their ability to detect if a genome is "contained" in a synthetic microbiome community metagenome. The authors' summary of this comparison is that both methods are equally accurate (though some differences in the third significant digit of correlation coefficient are mentioned, and their practical value unclear). Moreover, it is hard to do a comparison between the top and bottom rows of the figure (the two methods) when the x-axis are not comparable. As shown, each panel presents how a containment score varies with a resemblance score from the same method; a comparison of the containment scores (y-axis) may have been more intuitive if a common resemblance score was used on the x-axis, which may be possible in the simulated data using simulation parameters.

4)	A convincing advantage of kssd seems to be its lower running time for containment analysis, especially since costs of creating the initial sketches can be amortized over additional reference genomes to analyze in the future. On a more practical level however, I do not know if the running time improvements over say "mash screen" matter. In the context of the evaluations presented here, it seems that in the future the running time of ~2 hours can be saved when analyzing ~140,000 new genomes. What does this mean in practice? Why should we worry about saving ~2 hours of CPU time? Is it the case that people routinely analyze thousands of community metagenomes, in which case that run-time savings would translate to thousands of hours?

5)	Kssd is shown to perform all-pairs distance computation of 100,000 bacterial genomes in ~10 times less running (elapsed) time than the next-fastest method "bindash". This 10-fold speedup is actually a two-fold speedup when CPU time is considered, suggesting that I/O characteristics of the two tools may be the reason for the major speedup. The authors should comment on the interpretation of CPU-time speedup and elapsed time speedup, for the non-specialist reader.

6)	Figure 5 and its associated analysis presents an interesting case study of representing genomes from population studies such as the 1000 Genomes Project using kssd sketches and visualizing them using MDS plotting after all-pairs distance calculation with kssd. The run time and storage savings of such an analysis are impressive. However, the specific example of "HG01885" mislabeling was hard to follow, and the concluding sentence "Therefore, sample HG01855 in PRJEB31736 should be HG01885" remained puzzling (sounds like "X should be X").

7)	The sketching algorithm is reasonably intuitive, although the effect of non-uniformity of k-mer frequency distributions in different genomes was not clear.

Minor comments:

Line 202,232: "fastq-dumpped" -> "fastq-dumped"

Line 229: "the main cost lied in" -> "the main cost lay in"

**Authors Response**

**Point-by-point responses to the reviewers’ comments:**

Reviewer #1:

The authors developed a sketching technique to calculate the resemblance (similarity) and containment of two DNA sequences. This can be done by generating the set of all k-mers (substrings of length k) from each sequence, and then computing the Jaccard (resemblance) and containment coefficients of the two sets. The authors improve the performance of this by creating sketches containing a much smaller number of recoded k-mers, and running the same set operations on these sketches. They do this by first selecting an unbiased sample s of the k-mer space S (all possible sequences of length k). The sketch of a sequence contains only the k-mers of the sequence that are in s. The authors tested dimension reductions up to where s is 16^-4 the size of S (keeping about 1/65536 of the k-mers). The recoding step reduces the length of the k-mers by leveraging the fact that they are all in s. They demonstrated several applications for their algorithm, such as detecting incorrectly labeled samples, in large datasets. Although computation on the sketches is orders of magnitude faster than other algorithms, when including the time to generate the sketches, the overall approach was 12-30 times faster and about 2 times more accurate. Though, subsequent analyses are much faster since the sketches have already been calculated.

*Response: We appreciate the comments from this reviewer.*

Minor Comments:

Comment: 1) The authors write that the k-mer substring selection pattern (e.g., "000010010000") is "pre-determined" for 12-mer analysis. How was this selected? What weight w should be used? What patterns should be used for k-mers and substrings of different lengths? Why not place the 1's at the end so that it is easier to construct p0(K) and p1(K)?

*Response: The example kssp ‘000010010000’ is only used to show the basic idea of kssd. We have simplified the kssp setting in current kssd version by using only symmetric kssp ‘0{x-y}1{y}1{y}0{x-y}’, where x and y are the half-length of k-mer and the half-length of kmer substring namely weight w, respectively. Namely, k = 2x and w=2y. 0{x-y} and 1{y} mean a 0s string of length x-y and 1s string of length y, respectively. User can run this command to set x (here set by -k 9), y (here set by -s 6) and dimensionality reduction level z (here set by -l 3):*

*Kssd shuffle -k 9 -s 6 -l 3 -o out*

*For current version of kssd, x, y and z should satisfy 12 > x > y > z +2 > 1. y is suggested to greater than z+2 to ensure the substring space after dimension-reduction is still large enough (greater than 16^2) for robustness consideration. The program will suggest the choice of x, y and z if above requirements are not met. When developing kssd, we intuitively prefer a symmetric kssp (while place the 1's at the end is not symmetric) though we have never tested asymmetric kssp yet. The reviewer raised a very good suggestion to place the 1's at the end to simplify the substring selection step, we will test and consider it in future version of kssd. We addressed all these questions in the revised “Method” Implementation section.*

Comment: 2) The notation seems to change throughout the manuscript, which makes it difficult to read. For example, the Kssd program has an input variable -s, which is the half-length of the k-mer substring. Line 419 calls this "m". Though, I think this is the same as "w" on line 349. On line 352, "m" is related to the dimension reduction and used to determine the number of partitions, N=4^m. The Kssd program has another variable -l, which is the rate of dimensionality reduction. However, instead of it being a power of 4, on line 421 it is a power of 16. Does this mean that N=4^(2*l)? In the Figure 1 caption, they use lower-case "n" for the dimensionality-reduction levels (0 to 4), which I assume means N=16^n. Equation 20 also uses "m", but for something completely different.

*Response: We are sorry for the confusion caused. We have replaced these notions to improve readability in the revised manuscript. Specifically, line 419 m in original version, (Kssd -l variable) is replaced by y, it is a half of w ,so w = 2y; line 352 m is replaced by a, the n in the Figure caption (Kssd -l variable) is replaced by z, it is a half of a , so a=2z,N=4^a=4^2z (please also refer to the responses for last comment); m in Equation 20 is replaced by u.*

Comment: 3) The algorithm includes both dimension reduction and recoding. How much of the performance gain is due to each part? Is the performance benefit of recoding greater than the time needed to recode?

*Response: For the first question, the performance gained through recoding is due to dimension reduction per se. There is a tradeoff between performance (we mean time- and space-efficiency but not accuracy here) and accuracy: higher dimension reduction rate (or smaller sketch size) leads to higher performance gain but less accuracy for resemblance and containment measuring. Kssd is more accurate than mash and bindash when sketch size is the same (see Figure 3 in revised manuscript). This is achieved through both its sampling strategy and collisions-free recoding (as explained in “Introduction” and “Methods”). An accuracy threshold should be considered for most of practical applications. In these situations, kssd can uses higher dimension reduction rate (thus higher performance) than mash and bindash while still achieved similar accuracy. It means the performance gained through recoding as well as its sampling strategy is essentially due to dimension reduction, so we think the performance gain cannot be divided into due to dimension reduction part and due to recoding part.*

*For the second question, yes, the performance benefit of recoding is greater than the time needed to recode. Sketching methods have two stages: 1. sketching and 2. pairwise similarity/distance measuring. Recoding is only performed in stage 1. and does not affect stage 2. once stage 1. completed. Suppose the number of datasets under comparison is n, stage 1. spend O(n) time while stage 2. spend O(n2) time. Therefore, the performance is dominated by stage 2. but not stage 1., and the time needed for recoding is neglectable when n is large. Moreover, recoding itself is just rearranging k-mer string that needs only 7 operation by C programming in current version of kssd (6 bitwise operations + 1 array addressing operations), this is more efficient than the hash function used by other sketching methods for stage 1. And as explained in the first question, there is extra performance benefit translated from accuracy gain using recoding. Therefore, the performance benefit of recoding is greater than the time needed to recode.*

------------------------------------------------------------------------------

Reviewer #2:

In the manuscript under consideration, the authors presented Kssd, a novel method that compares two set of genomes using a novel design of sketch strategy. It is actually quite difficult to read in terms of both methods and results. I have read it carefully twice and finally figured out the idea behind the methods. I feel the idea is inspiring and deserves a publication. However, I still have some reservations on some of the main claims of the results and have some suggestions for authors to improve the presentation (assuming my understanding of their messages is correct). I list the items below for the authors' consideration:

*Response: We appreciate the reviewer’s positive comments on our idea. We apologize for not describing it clearly. We have done a lot editing to improve the readability in the revised manuscript.*

Claim that need to be strengthened:

Comment: 1) First, there is a claim that Kssd uses 5 magnitudes lower storage in Abstract. However, it appears they used the size of the sketch set of Kssd to compare the size of raw data used by Mash. This is an unfair comparison. To use Kssd, one doesn't need to download raw data at all?

*Response: After carefully reconsideration, we agree this claim might be misleading in the context of the original manuscript. In the revised manuscript, we have rephrased this claim to include the scenario when 5 magnitudes storage saving will stand in “Results” (Line 204-207). Abstract has been largely reduced to meet the editorial requirement (maximal 100 words) and all the claims on the computational efficiencies is generally expressed as “significantly faster, more accurate and less storage-costing”. We also explain here that the claim of 5 magnitudes lower storage (and data transfer) stands when the remote database adopt kssd sketching and provide kssd sketches for the raw datasets. For examples:*

*1) The public sequence database (e.g. NCBI-SRA) provide kssd sketch for each dataset. Then a user try to perform containment analysis only need download the sketches from the database, so there will be 5 magnitudes storage (and data transfer) saving (using Shakya dataset as an example).*

*• Constructing new databases that accept only sketches from submitters (for sketch-based analysis with sketches from other submitters or public sequences). So there will be 5 magnitudes storage (and data transfer) saving (using Shakya dataset as an example) for the remote database disk comparing to other databases accept raw data.*

*We understand these specific applications are not currently exist, but we believe these potential applications will be very useful in the near future.*

Comment: 2) Second, there are two cool results from the real data analyses: the identification of incorrectly registered species in metagenomic data, and the incorrectly labeled human samples. I am curious whether this is the advantage of Kssd? It appears to me that other methods may also identify such mistakes using contamination analysis?

*Response: Yes, both identification of incorrectly registered species in metagenomic data, and the incorrectly labeled human samples showed the advantages of Kssd. For the first case, kssd’s main advantage is its speed. Of course users can also use mash screen, but it will be much slower than kssd (Figure 4f). For the latter case, as far as we know, Kssd is currently the only method for large-scale datasets clustering/mislabeling detection/identification at population resolution. Other sketching methods has limited power for datasets comparisons at population resolution (Figure 7f). This is because population resolution datasets clustering/mislabeling detection/identification can only be achieved through reference subtraction (Figure 7f-h) which was first-time introduced by Kssd in this study (see Discussion Line 353-359). And non-sketching methods is too slow for large-scale datasets analyses (Figure 7e).*

Presentation:

Comment: 3) First of all, the inspiring idea that convinced me this is a great piece of work, depicted in Figure 6, may be better presented and presented upfront. I feel that the authors might explain the idea in Introduction and describe the algorithm in the first section of Results. They may want to move Figure 6 to Figure 1 so that the readers get their idea upfront.

*Response: We appreciate this reviewer’s positive comment and we think this is a very good suggestion. We have revised the manuscript as you suggested. Due to the limited legend space, Figure 6 now is split to two figures and moved upfront, Figure 1 explain the idea in Introduction (Line 130-134) and Figure 2 shows the algorithm overview in first section of Results (Line 149-160). The detailed description of the algorithm provides the necessary context to understand other parts of “Methods”, so is still kept in “Methods”.*

Comment: 4) Second, the motivation of the mathematical proofs in the Method section are not clear. What are their roles in the method development?

*Response: The motivation of Mathematical proofs is to justify the statistical properties of kssd. Firstly, we proved the orders of Kssd sketching operation and set operations (e.g. union, intersection and subtraction) are interchangeable, therefore the set operations on sketches is safe and accuracy-it equivalents to first do set operations on the raw datasets and then perform sketching on that resulting raw datasets (but much more efficient). Secondly, we proved kssd distance estimation is unbiased and given the calculations of its CI, p-value and Q-value which is very useful to measure uncertainty of the distance estimation. Lastly, we proved the properties of k-mer set decomposition and shows it is very usefully when ground truth J and C is needed in a memory-limited computer. All these has been stated in the revised manuscript.*

Comment: 5) Third, the claim of identifying incorrect labelled samples are unclear for me. If I understood the results correctly, the authors have aligned the reads files to the VCF-file supported reference genome and identified inconsistency? So, the reads files are still the standard and the VCF files are mislabeled? Please clarify this result.

*Response: We have clarified these concerns in the revised manuscript (see Line 289-302) and also explain it here: For both the analyses of genotype PCA and QTLtools mbv, yes, we aligned the reads files of sample HG01855 of PRJEB31736 to the 1KG VCF-file supported reference genome. For genotype PCA, we call genotype for HG01855 and combined HG01855 genotype with 2,504 1KG genotypes (total 2,505 samples), then performed genotype PCA for the 2,505 samples. We found HG01855 of PRJEB31736 are closest to HG01885 (but not HG01855) of 1KG (Fig. 7b), this confirmed that HG01855 of PRJEB31736 was mislabeled (discovered by kssd-mds in Fig. 7a). Since PRJEB31736 is just the resequencing project of the 2,504 1KG samples, the real label for HG01855 of PRJEB31736 must be one of the 2,504 samples other than HG01855. To find out the real label for HG01855, we compare the HG01855 bam file (generated by mapping all runs from HG01855 to the chromosome 1 reference) to 1KG VCF file using QTLtools mbv. And it shows the HG01885 is the real label. For both genotype PCA and QTLtools mbv, we used the 1KG VCF file (genotypes for all 2,504 1KG samples) as the golden standard to test HG01855 of PRJEB31736, so No, we think 1KG VCF file has no mislabeling. So why we test HG01855? From the very beginning, we aimed only to test the effectiveness of kssd-mds clustering on population-level. HG01855 is discovered to be population inconsistency by accident which may be explained by mislabeling (Figure 7a). Therefore, confirmation of HG01855 mislabeling event became a must to justify the effectiveness of kssd-mds clustering. We confirmed the mislabeling is real using genotype PCA and then found out the real label name for HG01855 of PRJEB31736 using both QTLtools mbv and kssd.*

Comment: 6) Fourth, there is a striking claim that the speed is 2500 times faster than state-of-the-art. However, this is not consistent with the data shown in Figure 3, the main display on CPU time. I carefully inspected the numbers and understood that Figure 3 is depicting something else different to the numbers they claimed in the abstract. I feel the authors should use a main display for their main claim in the abstract, instead of displaying other different calculations.

*Response: Thank for your suggestions. In the revised manuscript, we used an additional panel (Figure 4f) to display the 2500 times faster efficiency as it is a part of the result of the containment analysis. “Abstract” has been largely reduced to meet the editorial requirement (maximum 100 words) and all the claims on the computational efficiencies is generally expressed as “significantly faster, more accurate and less storage-costing”. This new claim “significantly faster” is supported by both Figure 3 (now Figure 5) and the containment analysis result (now Figure 4).*

Comment: 7) Fifth, there is a claim that "There is no need for Kssd to sketch the raw datasets again for the containment-analysis for new references". I can't understand why this is the case. When new references are added, we still need to generate the sub k-mer space for the new reference?

*Response: Sorry for the confusion caused. “the raw datasets” here is referred to “shakya datasets” which has been already sketched, and kssd performs containment analysis using sketches directly, so there is no need for Kssd to sketch the shakya datasets again. In the revised manuscript, we changed “raw datasets” here to “shakaya datasets” to improve the readability (Line 209). For new references, yes, we need to sketch the new references but the time is neglectable. In the revised manuscript, we have rephrased this claim to include the scenarios where this claim stands (Line 212-215). We also explain it here: When performing all SRA datasets (>1 million) against all new references (here, 138,743) containment analysis, the sketching time for the references is neglectable comparing to the sketching time for 1M raw datasets, therefore the asymptotic time-consumption of Kssd is only 1/ 2523 of that of Mash screen (3 CPU seconds vs. 126 CPU minutes).*

Comment: 8) Sixth, it is claimed that the method is hash-free. I feel this is not necessary (if not incorrect). Kssd still uses hash however choose to hash a subset of the full k-mer space instead of a subset of the genomic sequences (that is species-specific). This is an excellent idea but why calling if hash-free? I have no problem if the authors really want to call it that way: but it adds a bit difficulty to understand what exactly the algorithm is.

*Response: Thank you for your kindly reminder. To improve readability, we changed hash-free to (hash) collision-free in the revised manuscript. Kssd uses a one-to-one mapping from k-mer to integer in contrast to other methods using hash. Hash is a many-to-one function which would cause hash collision when two k-mers mapped to the same integer, Kssd totally avoids hash collisions.*

Comment: 9) Finally, why use line-chart in Figure 4? If the species depicted are different and we don't really expect to learn a trend, should we just use a bar-chart? Also, my understanding is that this figure plots the selected samples with incorrect registered references. If so, why are there a couple of outliers, SRR6045040 and ERR2789389)? Did I misunderstand your results?

*Response: Thank you for your suggestion. It is a good idea. We have replaced original Figure 4 using bar-chart (now Figure 6) in the revised manuscript. SRR6045040 is the only run with lower reads mappability on the overall nearest reference than on the registered specie reference, a possible explanation could be that the overall nearest reference was poorly assembled which hampered the reads mapping. ERR2789389 actually has higher mappability on the overall nearest reference than on the registered specie reference, but majority of the reads cannot be mapped to both references, suggesting there is severe contaminations from unknown species. We have provided the possible explanations in the revised manuscript.*

------------------------------------------------------------------------------

Reviewer #3

Yi et al. present a new, intuitive algorithm for representing a genome or genomic sequence data set in a highly compressed format (a "sketch") such that whole-genome comparisons can be performed very fast. These comparisons include measuring overall similarity of two genomes and detecting if a genome is "contained" in a metagenome. The authors demonstrate that the new tool (called kssd) is much faster and/or more storage-efficient than state-of-the-art in certain situations.

An impressive analysis is the detection of "species inconsistency", where a bacterial WGS data set is examined for containment of every bacterial reference genome and the best contained genome is found to be different from the species that the original data set was registered as. What is impressive is that over a million data sets are analyzed for containment of all prokaryotic genomes, resulting in over 100 billion containment coefficients, in only ~3 hours on a common 12 CPU machine. The species inconsistencies discovered are further confirmed as being real, although they are relatively rare (only ~6000 out of the ~1 M data sets, i.e., ~0.6%).

General comment: The authors present various evaluations and comparisons, and each highlights some positive aspects of the new tool, but also raises some questions about practical implications of those advantages. (See specific comments #2-4 below.) Perhaps the authors could highlight the most important application among these that they believe should convince the reader to adopt the new tool in place of the best alternatives. Since the main advantage overall is increased speed and/or decreased storage, the authors should try to argue that some task that *users will commonly want to perform* using sketches is *impractical currently* (e.g., will take days or months) but *can be made practical using kssd*. Without such a blunt argument, the merit of improved speed/storage is harder to appreciate.

*Response: We appreciate this reviewer for these suggestions. In the revised manuscript, we highlight in “Abstract” and “Results” that kssd is the only method for large-scale population resolution datasets clustering/mislabeling detection/correction. Other sketching methods do not have enough power for population resolution datasets clustering/mislabeling detection/correction. And non-sketching methods are impractical for large-scale datasets comparisons (Figure 7e). Therefore, large-scale population resolution datasets comparison is the most important application of Kssd.*

Specific comments:

Comment: 1) To someone not familiar with sketching tools, the introduction does a poor job of explaining what exactly the goal is. Existing sketching techniques are introduced and critiqued (e.g., lines 65-75) but it is hard to appreciate those criticisms if one doesn't know exactly which biological tasks will utilize the sketching and how those biological tasks are impacted by the supposed weaknesses.

*Response: We thank the reviewer for this suggestion. In the revised manuscript, we claim in the introduction the goal of Kssd is to answer “what reference genomes contained in which run?” and to address the hash-collision problem (Line 127-130); we also clarified that the biological tasks will utilize the sketching is “clustering of datasets, composition analysis of metagenomics and searching large-scale datasets for a certain sequence, etc.” and it “play an essential part in long-reads assembly and mapping” (Line 107-111). These below biological tasks are impacted by the supposed weaknesses: “datasets searching, distance estimation and clustering etc. (Line 118-119) and “prioritizing the references for a metagenomics dataset, or composition analysis of a sequences-mixture dataset, etc.” (Line 121-122).*

Comment: 2) Figure 1 is meant to show that mutation rates estimated using kssd are better than those estimated using existing methods mash and bindash. However, it is not clear what is the practical value of an improvement in the fourth significant digit of the correlation coefficient between predicted and "real" mutation rates.

*Response: We clarified this concern in the revised manuscript (Line 178-181), the accuracy (indicated by the correlation coefficient) can be translated to speed and storage-efficiency in practical applications. Figure 1 (now Figure 3 in revised manuscript) shows that generally users can use smaller sketches by kssd than by other methods, given the same accuracy (r) threshold. Supposing an application require accuracy threshold r = 0.96, this correspond y=-log(0.01) =3.3, Imaging a horizon line y=3.3 in Figure 3 across the curves, we can see, kssd, mash and bindash can use sketch size of roughly 84, 200 and 256 respectively to achieve the same accuracy threshold. This means kssd use < 1/2 sketch size of mash and bindash to achieve the same accuracy, when creating a sketch database, the size of the sketch database and the search speed by kssd is only 1/2 computational resource of other two sketching methods given their computational efficiency are the same (since the operations need for distance estimation is propositional to sketch size), this is an extra speed up, since kssd is much faster than others even if use similar sketch size ( as illustrated in Fig. 5)*

Comment: 3) Figure 2 is meant to compare kssd and an existing method "mash screen" for their ability to detect if a genome is "contained" in a synthetic microbiome community metagenome. The authors' summary of this comparison is that both methods are equally accurate (though some differences in the third significant digit of correlation coefficient are mentioned, and their practical value unclear). Moreover, it is hard to do a comparison between the top and bottom rows of the figure (the two methods) when the x-axis are not comparable. As shown, each panel presents how a containment score varies with a resemblance score from the same method; a comparison of the containment scores (y-axis) may have been more intuitive if a common resemblance score was used on the x-axis, which may be possible in the simulated data using simulation parameters.

*Response: We are sorry for the confusion caused. Figure 2 (Figure 4 in the revised manuscript) is meant to show kssd has the same ability as mash screen to detect the contaminations and low abundance constitutes-the outliers in the right panels. The related main text then shows kssd can achieve this using much less computational resources (our main points and one of our goals mentioned in “Introduction” of the revised manuscript Line126-130).*

*Figure 2 is a two-tier hierarchical experimental design: First-tier is the comparisons between the simulated shakaya dataset and the real shakaya dataset (or right panels and left panels). The second-tier is the comparison between kssd and mash screen (or top panels and bottom panels). The first-tier design is meant to show the contaminations and low abundance constitutes could be found as outliers in the left panels by the two methods independently. Therefore, both y-axis and x-axis should be measured by the same method alone without known ground truth (i.e. simulation parameters), otherwise its ability for outliers detection cannot be proved. The second-tier design is meant to compare the ability for outlier detection between kssd and mash screen, this can be partially demonstrated by the linearity between the resemblance and containment measurements of the same method. The first-tier design is totally from Ondov et.al’s mash screen paper, and Ondov et.al’s result was faithfully reproduced (show as the bottom panels) using the same procedure provided in their paper. The same procedure was also performed again using kssd instead of mash screen (top panels). Such designment is to demonstrate that kssd can replace mash screen for this kind of analysis by providing whatever mash screen can provide while using much less computational resources (shown in the main text and Figure 4e and f).*

Comment: 4) A convincing advantage of kssd seems to be its lower running time for containment analysis, especially since costs of creating the initial sketches can be amortized over additional reference genomes to analyze in the future. On a more practical level however, I do not know if the running time improvements over say "mash screen" matter. In the context of the evaluations presented here, it seems that in the future the running time of ~2 hours can be saved when analyzing ~140,000 new genomes. What does this mean in practice? Why should we worry about saving ~2 hours of CPU time? Is it the case that people routinely analyze thousands of community metagenomes, in which case that run-time savings would translate to thousands of hours?

*Response: We agree that a 2-hours saving is a trivial improvement for users only need analysis single sample. While kssd was developed mainly for large-scale datasets analysis. As clarified in the introduction of the revised manuscript, one of the goals of Kssd is to answer the question “what genomes in which runs”(Line126-130). We concern more about the computational efficiency when sample size is large. With the continuously drop of sequencing-cost, metagenomes sequencing project of larger sample size is the trend, so tools suite for large-scale datasets analysis like kssd should be more and more popular in the near future. We have revised this part to include the scenarios where kssd has its main advantages (Line 212-216)*

Comment: 5) Kssd is shown to perform all-pairs distance computation of 100,000 bacterial genomes in ~10 times less running (elapsed) time than the next-fastest method "bindash". This 10-fold speedup is actually a two-fold speedup when CPU time is considered, suggesting that I/O characteristics of the two tools may be the reason for the major speedup. The authors should comment on the interpretation of CPU-time speedup and elapsed time speedup, for the non-specialist reader.

*Response: Thank you for your suggestion. We think this phenomenon could cause by a more optimized parallel design or a better I/O characteristic of Kssd. We provide the interpretation in the revised manuscript as you suggested (Line 235-237).*

Comment: 6) Figure 5 and its associated analysis presents an interesting case study of representing genomes from population studies such as the 1000 Genomes Project using kssd sketches and visualizing them using MDS plotting after all-pairs distance calculation with kssd. The run time and storage savings of such an analysis are impressive. However, the specific example of "HG01885" mislabeling was hard to follow, and the concluding sentence "Therefore, sample HG01855 in PRJEB31736 should be HG01885" remained puzzling (sounds like "X should be X").

*Response: Thank you for your kindly reminder for the readability problem of this part of manuscript. If we have understood the reviewer’s confusion correctly, the reviewer might have thought HG01855 and HG01885 were the same thus lead confusion? Indeed, the two label names look very similar which may explain why it was mislabeled. In the revised manuscript, we have improved the readability of this part and highlighted the difference of HG01855 and HG01885 (Line 311).*

Comment: 7) The sketching algorithm is reasonably intuitive, although the effect of non-uniformity of k-mer frequency distributions in different genomes was not clear.

*Response: Kssd performs a random k-mer (or its substring) space sampling that is independent of any given genomes. Theoretically, any k-mer in any input genomes has equal probability to be drawn regardless of the specific k-mer frequency of a given genome.*

Minor comments:

Comment: 8) Line 202,232: "fastq-dumpped" -> "fastq-dumped"

*Response: We appreciate the reviewer and we have corrected the typo in the revised manuscript.*

Comment: 9) Line 229: "the main cost lied in" -> "the main cost lay in"

*Response: We appreciate the reviewer and we have corrected the typo in the revised manuscript.*

**Second round of review**

**Reviewer 2**

The revised manuscript reads well. All my comments are generally addressed.

**Reviewer 3**

The authors have done a commendable job addressing all of my previous doubts and suggestions.
